# Short-Term TERT Inhibition Impairs Cellular Proliferation via a Telomere Length-Independent Mechanism and Can Be Exploited as a Potential Anticancer Approach

**DOI:** 10.3390/cancers15102673

**Published:** 2023-05-09

**Authors:** Aamir Amin, Marzia Morello, Maria Raffaella Petrara, Beatrice Rizzo, Francesco Argenton, Anita De Rossi, Silvia Giunco

**Affiliations:** 1 Department of Surgery, Oncology and Gastroenterology, Section of Oncology and Immunology, University of Padova, 35128 Padova, Italy; aamir.amin@studenti.unipd.it (A.A.); raffaella.petrara@unipd.it (M.R.P.); anita.derossi@unipd.it (A.D.R.); 2 Immunology and Diagnostic Molecular Oncology Unit, Veneto Institute of Oncology IOV—IRCCS, 35128 Padova, Italy; marzia.morello@iov.veneto.it (M.M.); beatrice.rizzo@iov.veneto.it (B.R.); 3 Department of Biology, University of Padova, 35128 Padova, Italy; francesco.argenton@unipd.it

**Keywords:** TERT/telomerase, TERT extra-telomeric functions, NF-kB p65, MYC, EBV-immortalized and fully transformed B cells, new therapeutic approach, zebrafish, xenograft

## Abstract

**Simple Summary:**

Blocking telomerase to drive telomere erosion-dependent antiproliferative effects in cancer cells appears impractical. However, the evidence of extra-telomeric functions of TERT, the catalytic component of telomerase, in promoting tumour growth/progression strongly supports the potential telomere length-independent therapeutic effects of TERT inhibition. The mechanism(s) underlying these effects need to be explored to identify cellular pathways being (de)regulated by telomerase during the oncogenic process to establish how the selective targeting of TERT can rapidly interrupt the expansion of tumour cells, regardless of telomere length and erosion. Using in vitro models of B-cell lymphoproliferative disorders and B-cell malignancies, we found that TERT inhibition impairs the NF-κB p65 pathway, resulting in decreased MYC expression and a consequent P21-mediated cell cycle arrest. The in vivo results in the zebrafish model confirm the in vitro data and prompt an evaluation of strategies combining TERT inhibition with chemotherapeutic agents to enhance the therapeutic benefits of current treatment modalities.

**Abstract:**

Telomerase reverse transcriptase (TERT), the catalytic component of telomerase, may also contribute to carcinogenesis via telomere-length independent mechanisms. Our previous in vitro and in vivo studies demonstrated that short-term telomerase inhibition by BIBR1532 impairs cell proliferation without affecting telomere length. Here, we show that the impaired cell cycle progression following short-term TERT inhibition by BIBR1532 in in vitro models of B-cell lymphoproliferative disorders, i.e., Epstein-Barr virus (EBV)-immortalized lymphoblastoid cell lines (LCLs), and B-cell malignancies, i.e., Burkitt’s lymphoma (BL) cell lines, is characterized by a significant reduction in NF-κB p65 nuclear levels leading to the downregulation of its target gene MYC. MYC downregulation was associated with increased expression and nuclear localization of P21, thus promoting its cell cycle inhibitory function. Consistently, treatment with BIBR1532 in wild-type zebrafish embryos significantly decreased Myc and increased *p21* expression. The combination of BIBR1532 with antineoplastic drugs (cyclophosphamide or fludarabine) significantly reduced xenografted cells’ proliferation rate compared to monotherapy in the zebrafish xenograft model. Overall, these findings indicate that short-term inhibition of TERT impairs cell growth through the downregulation of MYC via NF-κB signalling and supports the use of TERT inhibitors in combination with antineoplastic drugs as an efficient anticancer strategy.

## 1. Introduction

Telomerase is a ribonucleoprotein complex composed of two core components: a non-coding telomerase RNA (telomerase RNA component, TERC) and a catalytic subunit (telomerase reverse transcriptase, TERT) with reverse transcriptase activity for telomeres. Telomeres are repetitive (TTAGGG) DNA structures present at the end of chromosomes, essential for maintaining the genomic integrity of cells [1,2]. The main function of the telomerase is to compensate for the loss of telomeric ends, which occurs during each cell division because of end-replication problems in DNA polymerase [3]. Thus, TERT, by stabilizing telomere length, prevents cell senescence and apoptosis. TERT is the rate-limiting component of the telomerase complex [4], and its expression, usually absent in normal somatic cells, is detectable in ~90% of human malignancies [5], allowing cancer cells to overcome the replicative crisis caused by telomere attrition [6,7]. In addition to its role in telomere maintenance, a growing body of evidence has ascribed various telomere length-independent functions to this enzyme [8]. These functions include regulation of gene expression [9], enhancement of cell proliferation kinetics [10], modulation of DNA damage responses (DDR) [11,12], and resistance to apoptosis [13,14], all of which can contribute to tumour formation and progression.

BIBR1532 (BIBR) is a non-competitive non-nucleoside small molecule that selectively inhibits telomerase catalytic activity by binding to a hydrophobic pocket, conserved across species, on the superficial region of TERT, preventing proper telomerase ribonucleoprotein assembly and enzymatic activity [12,15,16,17,18,19,20]. In particular, BIBR binds to the N-terminal domain (TEN) of TERT close to the enzyme catalytic core and blocks its conformation in a closed state, consequently disturbing the active loop conformation and the enzyme processivity [16]. Our previous in vitro studies demonstrated that short-term telomerase inhibition by BIBR impaired cell proliferation with an accumulation of cells in the S-phase and induced apoptosis associated with the activation of DDR via a telomere length-independent mechanism, both in Epstein-Barr virus (EBV)-immortalized lymphoblastoid cell lines (LCL) and in Burkitt lymphoma (BL) cells [12]. Moreover, TERT inhibition by BIBR in LCL cells enhanced the pro-apoptotic and anti-proliferative effects of fludarabine (FLU) and cyclophosphamide (CY), two chemotherapeutic agents frequently used to treat B-cell malignancies [12]. Recently, we demonstrated that short-term telomerase inhibition, without effects on telomere length, negatively impacts cell proliferation and viability, both in the in vivo system and in human malignant B cells xenografted in zebrafish [21].

The molecular mechanism(s) of how telomerase plays an oncogenic role beyond telomere maintenance are still subject to debate. Some studies suggest that TERT interacts with transcription factors such as the RELA proto-oncogene, NF-kB subunit (NF-κB, p65) [22,23,24,25,26,27,28], MYC proto-oncogene, bHLH transcription factor (MYC) [29,30], catenin beta 1 (CTNNB1, β-catenin) [31,32] or SWI/SNF related, matrix-associated, actin-dependent regulator of chromatin, subfamily a, member 4 (SMARCA4, BRG1) [33], depending on the cellular context, and modulates gene expression. On the other hand, these transcription factors can drive *TERT* promoter activity [34,35,36,37], establishing a feed-forward signalling loop. It has been proposed that there is a link between TERT, NF-κB p65, and MYC transcriptional programs given that withdrawal of TERT, likely by impairing the binding of p65 and MYC to their target promoters, alters the expression of their related genes [23,24,29], thus suggesting that TERT works as a transcriptional amplifier in cancer.

In cancer, telomerase reactivation often exists in parallel with MYC overexpression [38]. Notably, MYC is involved in oncogenic processes through the activation of pro-tumorigenic genes, including TERT [34,39]. In turn, TERT has been shown to have a direct role in the MYC pathway, either by regulating the wild-type *MYC* promoter by binding the MYC transcription factor NME/NM23 nucleoside diphosphate kinase 2 (NME2) [40] or through its interaction with MYC at the protein level [29,30]. MYC is essential for the proliferation of both immortalized LCL and BL cells, where MYC downregulation leads to an S-phase cell cycle arrest [41].

TERT has also been shown to functionally intersect with NF-κB signalling. NF-κB is a well-known transcriptional factor and is involved in the transcriptional regulation of both wild-type and translocated *MYC* promoters [42,43] and can drive *TERT* promoter activation either directly [34] or through transcriptional activation of *MYC* [35,44]. On the other hand, it has been demonstrated that TERT can directly regulate NF-κB p65 nuclear levels, with the subsequent activation of a subset of NF-κB target genes [23,24]. Consistently, silencing of TERT or chemical telomerase inhibition reduces p65-mediated transcription of NF-κB target genes [23,24,25,26].

Currently, no data is available on the mechanism(s) involved in the cell cycle arrest induced by TERT inhibition in EBV-immortalized and fully transformed B cells. In the present study, we analysed the mechanisms involved in the non-canonical functions of TERT. In this regard, we studied the interactions of TERT with NF-κB p65 and MYC in in vitro LCL and BL cell lines. Furthermore, we also explored the mechanisms through which Tert inhibition affects cell proliferation in an in vivo zebrafish model. Zebrafish (Danio rerio) proved to be a useful model for studying several areas of cancer research [45], including the characterization of the non-canonical functions of Tert [21,46]. The availability of zebrafish telomerase mutants (*tert^hu3430/hu3430^* or *tert−/−*) [47] also makes this model relevant to studying in vivo the specific impact of telomerase-targeted therapies. Furthermore, the zebrafish model serves as a bridge between in vitro assays and mammalian in vivo studies [48] and is considered a valuable in vivo tool for preliminary drug screening [49]. Therefore, in light of the possible integration of TERT inhibitors in chemotherapeutic regimens, we evaluated the possible therapeutic application of the combined treatment with TERT inhibitors and antineoplastic drugs frequently used to treat B-cell malignancies to counteract tumour growth in vivo.

## 2. Materials and Methods

### 2.1. Compounds

A stock solution of BIBR (Selleck Chemicals LLC, Houston, TX, USA) at a concentration of 50 mM was prepared by dissolving the compound in sterile dimethyl sulfoxide (DMSO) and stored in small aliquots at −80 °C until use. Ammonium pyrrolidine dithiocarbamate (PDTC) (P8765; Sigma-Aldrich, Saint Louis, MO, USA) was prepared by resuspending the compound in sterile water at a concentration of 10 mM divided into aliquots and stored at −20 °C until use. Fludarabine (FLU, F9813; Sigma-Aldrich) was prepared by resuspending the compound in DMSO at a concentration of 10 mM. Cyclophosphamide (CY, 0768; Sigma-Aldrich) was prepared by dissolving the compound in sterile water at a concentration of 143.3 mM.

### 2.2. Cell Cultures

The 4134/Late LCL was derived from late passages of peripheral blood mononuclear cells from a normal donor infected with the B95.8 EBV strain and expresses high endogenous levels of TERT [12,50]. In agreement with Faumont et al., we employed this cell line as an in vitro model of EBV-driven post-transplant lymphoproliferative disorders (PTLD) [41]. BL41 is an EBV-negative Burkitt’s lymphoma cell line with translocated *MYC* gene (kindly provided by Martin Rowe, Cancer Centre, University of Birmingham, Birmingham, UK). Protein expression and mRNA levels of TERT and telomerase activity have already been evaluated in these cell lines [12,50,51]. BIBR efficiently inhibits telomerase activity in these cell lines [12]. LCLs and BL41 cells were cultured in RPMI-1640 medium (Euroclone, Milan, Italy), supplemented with 4 mM L-glutamine, 50 mg/mL gentamycin (Sigma-Aldrich) and 10% heat-inactivated fetal bovine serum FBS (Gibco, Milan, Italy). The human osteosarcoma cell line (U2OS) was obtained from the American Type Culture Collection (Rockville, MD, USA) and was maintained in McCoy’s 5A modified medium (Thermo Scientific, Waltham, MA, USA), supplemented with 50 mg/mL gentamycin and 10% FBS (Gibco). All cell lines used were maintained in culture at 37 °C in a 5% CO_2_ incubator and tested negative for mycoplasma contamination.

### 2.3. Animals

All experiments were performed in accordance with European and Italian legislation and with permission for animal experimentation from the Local Ethics Committee of the University of Padova and the Italian Ministry of Health (protocol numbers 569/2018-PR and 259/2020-PR). Zebrafish were maintained in a temperature-controlled (28.5 °C) environment and fed as described by Kimmel et al. [52].

For Tert inhibition experiments, wild-type (WT) and *tert* mutant (*tert^hu3430/hu3430^*; *tert−/−*) zebrafish embryos were treated at the stage of 12 h post-fertilization (hpf), when Tert expression is high in WT zebrafish embryos [46], with 2 µM BIBR or DMSO as a control, and samples were analysed after 12 h of treatment, i.e., at 24 hpf. Two µM of BIBR has been demonstrated to reduce telomerase activity in WT zebrafish [21] and the treatment has proven to be effective in halting viability and proliferation in WT embryos without affecting the telomerase-negative ones employed as controls [21]. The telomerase mutant zebrafish line (allele *tert^hu3430^*) has already been described [47] and no evidence of telomerase activity was observed in protein extracts from *tert−/−* zebrafish samples [21,47].

The transparent casper strain (*mpv17−/− mitf−/−*) of zebrafish was employed for the xenotransplantation experiments (see below).

To test the highest tolerable dose of the chemotherapeutic agents (CY and FLU) that does not alter zebrafish viability, 72 hpf casper zebrafish embryos were exposed to different doses of FLU or CY, and viability was analysed after 72 h of treatment. As shown in Supplementary Figure S1, 8 µM FLU (Figure S1A) and both 2 and 4 mM CY (Figure S1B) significantly increased embryonic lethality compared to those in the untreated control embryos. Conversely, 5 µM FLU (Figure S1A) and 1 mM CY (Figure S1B) did not alter the viability of the embryos compared to the controls; thus, these concentrations were employed in the xenograft experiments.

### 2.4. Plasmids and Transfection

The plasmids employed were the following: a plasmid expressing human TERT (pBABE-hTERT) [50], a plasmid expressing a derivative of the human TERT protein with a hemagglutinin (HA) epitope tag to its C terminus (hTERT-HA), and the empty control vector (pBABE) (gifts from Bob Weinberg, Addgene, Watertown, MA, USA). The transfections were performed using Lipofectamine 2000 (Invitrogen, Carlsbad, CA, USA), according to the manufacturer’s instructions.

### 2.5. Reverse Transcription and Quantitative Real-Time PCR

Total cellular RNA was extracted from 5 × 10^6^ cells using 750 µL Trizol reagent (Invitrogen), according to the manufacturer’s instructions, and quantified using Nanodrop One (Thermo Scientific). For quantitative real-time PCR experiments, 1 µg RNA was retrotranscribed into cDNA using SuperScript III RNA Reverse Transcriptase (Invitrogen) following the manufacturer’s instructions. For the in vivo experiments, total RNA was extracted from 20 WT and *tert−/−* embryos treated for 12 h, from 12 to 24 hpf, with BIBR or DMSO. Embryos were manually dechorionated, collected in 1.5 mL tubes, and washed twice with phosphate-buffered saline (PBS). Seven hundred and fifty µL of Trizol reagent were added to each sample, and RNA was extracted and retrotranscribed into cDNA as described above.

Quantitative Real-time PCR reactions were performed in duplicate in Platinum SYBR Green qPCR SuperMix (Thermo Scientific) in an ABI PRISM 7900HT Sequence Detection System (PE Biosystems, Foster City, CA, USA). Hypoxanthine phosphoribosyltransferase 1 (*HPRT1*) and glyceraldehyde-3-phosphate dehydrogenase (*gapdh*) were employed as in vitro and in vivo internal controls, respectively. The amount of target gene, normalized to the housekeeping gene and relative to a calibrator (DMSO-treated sample), was given by the arithmetic formula: 2^–ΔΔCT^ [53]. TERT transcripts were quantified using the AT1/AT2 primer pair as previously described [50,54].

The sequences of the primers used for real-time PCR are listed in Table S1.

### 2.6. Immunoblot and Co-Immunoprecipitation

Whole-cell lysates were prepared in radioimmunoprecipitation assay (RIPA) buffer (Cell Signaling Technology, Danvers, MA, USA) containing 1× Halt protease and phosphatase inhibitor cocktail (Thermo Scientific) for 30 min, followed by centrifugation at 14,000 rpm. The proteins in the supernatants were harvested and quantified using the Pierce BCA protein Assay kit (Thermo Scientific).

For in vivo experiments, protein lysates were prepared from 50 WT and *tert−/−* embryos treated with BIBR or DMSO at 24 hpf, as previously described [21].

Equal amounts of proteins (30–50 µg) were separated on a 4–15% TGX gel (Bio-Rad, Hercules, California, USA) and blotted on nitrocellulose membrane (Ge Healthcare Life Sciences, Cytiva, Marlborough, MA, USA), using the Trans-Blot Turbo Transfer System (Bio-Rad). The expression of TERT, MYC, p65, phosphorylated p65 (p-p65), cyclin-dependent kinase inhibitor 1A (CDKN1A, P21), telomeric repeat binding factor 2 (TRF2), and α-tubulin were evaluated by the following antibodies: TERT (ab32020, Abcam, Cambridge, UK; abx120550, Abexxa, Arlington, TX, USA), NF-κB p65 (ab32536, Abcam), phospho-NF-κB p65 (Ser536) (93H1, Cell Signaling), MYC (ab32072, Abcam), P21 (ab109520, Abcam), TRF2 (NB110-57130, Novus Biological, Littleton, CO, USA), GAPDH (GTX100118, GeneTex, Alton Pkwy, Irvine, CA, USA), and α-tubulin (Sigma-Aldrich). Blots were incubated with appropriate peroxidase-conjugated secondary antibodies and stained with a chemiluminescent detection kit (SuperSignal West Pico Plus Chemiluminescent Substrate, Pierce, Thermo Scientific). For densitometric analysis, the signal was quantified using ImageJ software version 1.53e and normalised against appropriate housekeeping proteins.

For co-immunoprecipitation, cells were lysed in immunoprecipitation (IP) cell lysis buffer (9803, Cell Signaling) on ice, followed by 10 min of centrifugation at 14,000 rpm. The proteins in the supernatants were quantified using the Pierce BCA protein assay kit (Thermo Scientific). Immunoprecipitation was performed using 1 mg total proteins in 1 mL cell lysate. Following pre-clearing, antibodies were added following the manufacturer’s instructions and incubated overnight at +4 °C on a rotary mixer with gentle rocking. The next day, Protein A/G Sepharose (ab193262, Abcam) 50% bead slurry was added, and samples were incubated for 2 h at +4 °C with gentle rocking. Beads were harvested by slow speed centrifugation at +4 °C and washed five times with 1× cell lysis buffer. Following the final wash, immunocomplexes were eluted using 3× blue loading buffer (Cell Signaling). The eluted proteins were analysed by immunoblotting as described above.

### 2.7. Nuclear and Cytoplasmic Fraction

Subcellular fractionation was performed with NE-PER Nuclear and Cytoplasmic Extraction Reagent (Thermo Scientific) following the manufacturer’s instructions. Briefly, cells were collected in ice-cold PBS, suspended in CER-I buffer, and incubated on ice for 10 min. The CER-II buffer was added, and cells were vortexed for 5 s twice with a 1 min interval, followed by immediate centrifugation at 14,000 rpm for 5 min. The supernatant was collected as a cytoplasmic fraction in separate tubes. The cell pellet was lysed in NER buffer on ice for 40 min, followed by 15 s vortexing every 10 min, centrifugation at 14,000 rpm for 10 min, and the supernatant was collected as a nuclear fraction. The cytoplasmic and nuclear protein fractions were quantified and immunoblotted using the protocol described above.

### 2.8. Immunofluorescence

Cells treated with BIBR or DMSO as a control were harvested in ice-cold PBS at approximately 1 × 10^6^ cells/mL. Two mL cell suspension were added to each well of the P6 cell culture plate (P6) containing coverslip, and cells were allowed to attach through gravity sedimentation at 37 °C for 30 min, as previously described [55]. Following cell adhesion, PBS was slowly aspirated, and the attached cells were fixed in 10% formalin for 10 min at room temperature. Cells were washed in PBS for 5 min and then permeabilized for 10 min at room temperature using 0.5% Triton X-100 in PBS. Cells were blocked in 1% BSA for 30 min and incubated with rabbit monoclonal P21 (ab109520, Abcam) antibody following the manufacturer’s instructions, overnight at +4 °C, followed by three PBS washes at room temperature. The coverslips were then incubated with Alexa Fluor Donkey 488 anti-rabbit secondary antibody (Thermo Scientific) at room temperature for 1 h in the dark, washed three times, and cell nuclei counterstained with propidium iodide (PI) (1 µg/mL) for 10 min. Finally, coverslips were mounted inverted on clear glass slides using ProLong Gold Antifade Mountant (Thermo Scientific). Slides were air-dried for 15 min and then visualized using ZEISS LSM 900 with an Airyscan 2 confocal fluorescence microscope (Carl Zeiss Microscopy GmbH, Jena, Germany).

### 2.9. Cell Viability and Cell Cycle Analysis

Cell viability was determined by trypan blue cell exclusion using a Countess automated cell counter (Invitrogen). Cell cycle analysis of cells treated with either BIBR, PDTC, or DMSO was performed by PI staining, as previously described [12]. Samples were analysed using a FACS Calibur Flow Cytometer (BD Biosciences, Franklin Lakes, NJ, USA), and cell cycle distribution was measured using ModFit LT Cell Cycle Analysis software version 2 (Verity Software House, Topsham, ME, USA).

### 2.10. Telomere Length Measurement

DNA was extracted from 5 × 10^6^ cells using the QIAmp DNA Mini Kit (Qiagen, Hilden, Germany) according to the manufacturer’s instructions. Relative telomere lengths were determined by quantitative multiplex PCR assay, as described by Cawthon with a few modifications [56,57]. In particular, each PCR reaction was performed in a final volume of 25 μL, containing a 5 μL sample (10 ng DNA) and a 20 μL master-mix ready-to-use 1× Light Cycler 480 SYBR Green I (Roche Diagnostic, Mannheim, Germany), containing 900 nmol/L of each primer. The primer pair employed for telomere amplification were the following: TELG 5′-ACACTAAGGTTTGGGTTTGGGTTTGGGTTTGGGTTAGTGT-3′, and TELC 5′-TGTTAGGTATCCCTATCCCTATCCCTATCCCTATCCCTAACA-3′. The primer pair for amplification of single-copy gene albumin were the following: ALBU 5′-CGGCGGCGGGCGGCGCGGGCTGGGCGGAAATGCTGCACAGAATCCTTG-3′ and ALBD 5′-GCCCGGCCCGCCGCGCCCGTCCCGCCGGAAAAGCATGGTCGCCTGTT-3′. The thermal cycling profile was 15 min at 95 °C, two cycles of 15 s at 94 °C, and 15 s at 51 °C, followed by 40 cycles of 15 s at 94 °C, 10 s at 62 °C, 15 s at 74 °C, 10 s at 84 °C, and 15 s at 89 °C, with signal acquisition at the end of both the 74 °C and 89 °C steps. After cycling, a melting curve program was run, starting with a 95 °C incubation for 1 min, followed by continuous acquisitions every 0.2 °C for 45 °C to 95 °C (ramping at 0.11 °C/s). A standard curve was generated at each PCR run, consisting of DNA from the RAJI cell line serially diluted from 20 to 0.08 ng/μL. All DNA samples and reference samples were run in triplicate. LightCycler raw text files were converted using the LC480Conversion free software (http://www.hartfaalcentrum.nl/index.php?main=files&-fileName=LC480Conversion.zip&description=LC480Conversion:%20conversion%20of%20raw%20data%20from%20LC480&sub=LC480Conversion (accessed on 15 August 2012, version 2)), and the converted data were analysed using LinRegPCR free software version 2012.3.2.0 to obtain the Ct values. Mean Ct values were used to calculate the relative telomere length using the telomere/single-copy-gene ratio (T/S) according to the formula: ΔCt_sample_ = Ct_telomere_ − Ct_albumin_, ΔΔCt = ΔCt_sample_ − ΔCt_reference curve_ (where ΔCt_reference curve_ = Ct_telomere_RAJI_ − Ct_albumin_RAJI_) and then T/S = 2^−ΔΔCt^ [58].

### 2.11. Xenotransplantation of LCL and BL Cells in Zebrafish Embryos

Xenograft experiments were performed as previously described [21]. Briefly, approximately 300 4134/Late or BL41 cells, pre-treated for 16 h with 30 µM BIBR (pre-BIBR) or DMSO (pre-DMSO) as a control, were fluorescently labelled with the vital cell tracker red fluorescent chloromethylbenzamido derivative of octadecylindocarbocyanine (CM-DiI) (Invitrogen), followed by microinjection into the yolk sac of 72 hpf transparent casper zebrafish embryos that were subsequently transferred to 32 °C. Twenty-four h post-xenotransplantation (hpx), the embryos were selected according to the intensity of the engrafted mass; only embryos with similar fluorescence intensity were chosen, while non-fluorescent embryos or embryos with fluorescent cells outside the place of injection were discarded. The chosen xenografted embryos were divided into 6 experimental groups, and drugs were added to the medium as follows: pre-DMSO xenograft embryos without treatment (pre-DMSO NT), pre-DMSO CY embryos (pre-DMSO CY), pre-DMSO FLU embryos (pre-DMSO-FLU), pre-BIBR drugs unexposed embryos (pre-BIBR NT), pre-BIBR CY embryos (pre-BIBR CY), and pre-BIBR FLU embryos (pre-BIBR FLU). Zebrafish were kept at 32 °C until the end of the experiments. The percentage of labelled cells in the engrafted embryos was determined at 24, 48, and 72 h post-treatment (hpt) in enzymatically dissociated embryos by flow cytometric analysis (see below).

### 2.12. Embryo Dissociation and Flow Cytometric Analysis

The dissociation of zebrafish embryos in a single-cell suspension was performed as previously described [21]. Cell suspensions obtained from 10 embryos per condition were employed to monitor fluorescent cells for proliferation by flow cytometric analysis in an LSR II cytofluorimeter (Becton-Dickinson, San Jose, CA, USA). Xenografted human-labelled LCL or BL cells were detected based on the fluorescence intensity signal of the CM-DiI fluorochrome. Non-xenografted embryos, included in each experiment, were employed to set the threshold as previously described [21]. Data were processed with FACSDiva Software (Becton-Dickinson) and analysed using Kaluza Analyzing Software v.1.2 (Beckman Coulter, Fullerton, CA, USA).

### 2.13. Statistical Analyses

Statistical analyses were performed with Prism software version 9 (GraphPad Software Inc.; La Jolla, CA, USA). Results were analysed with the Student’s *t*-test, and *p*-values < 0.05 were considered statistically significant.

## 3. Results

### 3.1. TERT Inhibition Reduced Nuclear Levels of p65

**Figure 1:**
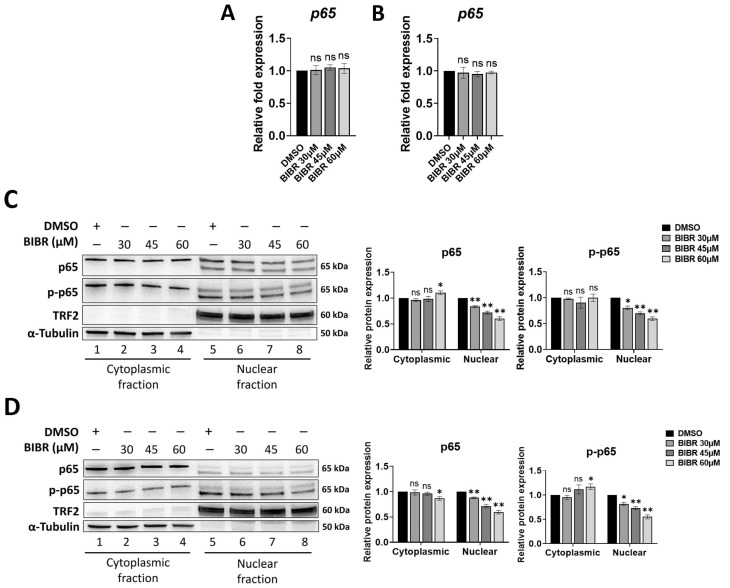
Telomerase inhibition altered RELA proto-oncogene NF-kB subunit (p65) nuclear levels. (**A**,**B**) Cells were treated with indicated concentrations of BIBR1532 (BIBR) or dimethyl sulfoxide (DMSO) as control, for 24 h. Levels of relative mRNA expression for *p65* in 4134/Late (**A**) and BL41 (**B**) cells are shown. Data represent the mean and standard deviation (SD, bar) from three separate experiments. (**C**,**D**) Cells treated with BIBR at indicated concentrations, or DMSO as control, for 24 h were processed to obtain cytoplasmic and nuclear extracts. Representative Western blots showing cytoplasmic and nuclear protein levels of p65, phospho-p65 (p-p65), telomeric repeat binding factor 2 (TRF2), and α-tubulin in 4134/Late (**C**) and BL41 (**D**) cells are shown. α-tubulin and TRF2 were used as loading controls for the cytoplasmic and nuclear fractions, respectively. The original Western blots are shown in File S1. Graphs next to the blots show the values in arbitrary units of densitometric analysis performed with ImageJ software. Data represent the mean and SD (bar) from three separate experiments. A significant difference between values in BIBR-treated vs. DMSO-treated cells is shown: * *p* < 0.05; ** *p* < 0.01; ns: not significant.

We have previously shown that short-term TERT inhibition by BIBR impairs cellular proliferation in in vitro models of post-transplant lymphoproliferative disorders (i.e., LCL cell lines) and Burkitt’s lymphoma (i.e., BL cell lines) without any detectable change in telomere length, thus suggesting a druggable extra-telomeric function of TERT involved in cellular proliferation [12]. Interestingly, a telomere-independent role of TERT has been demonstrated as a transcriptional modulator of the NF-κB signalling pathway [23,24,25,26]. NF-κB is a well-known transcriptional factor having critical functions in B-cell malignancies; in particular, enhanced proliferation of LCLs and BL cells was found to be dependent on NF-κB signalling as NF-κB inhibition decreased cellular proliferation in both cell types [59,60]. Therefore, to shed light on the possible mechanism underlying impaired proliferation upon short-term TERT inhibition, the effect of different doses (30, 45, and 60 µM) of BIBR treatment on NF-κB p65 expression was investigated in LCL and BL cells. Similar to the previous results obtained with 30 µM BIBR treatment [12], treatment with 45 or 60 µM BIBR also resulted in decreased proliferation rates, starting from 24 h of exposure, in both 4134/Late (Figure S2A) and BL41 (Figure S2B) cells. At 24 h of treatment, we previously observed a strong cell cycle arrest with an accumulation of cells in the S-phase [12]; therefore, we chose this time point for the subsequent analysis. In addition, short-term treatment with different doses of BIBR did not affect the telomere length of 4134/Late and BL41 cells, as measured by quantitative multiplex PCR at 24 h of exposure (Figure S2C,D). Twenty-four h of TERT inhibition by BIBR altered p65 protein levels without any change in *p65* transcription in both 4134/Late (Figure 1A) and BL41 (Figure 1B) cells. Particularly, BIBR significantly reduced p65 nuclear expression in a dose-dependent manner: from 17 ± 2% to 39 ± 2.5% at 30 μM and 60 μM, respectively, (*p* < 0.01), in 4134/Late cells (Figure 1C), and from 12 ± 1% to 40 ± 3% at 30 μM and 60 μM respectively, (*p* < 0.01) in BL41 cells (Figure 1D), without any significant change in its cytoplasmic levels. Interestingly, the BIBR treatment even reduced the phosphorylated active form of p65 (p-p65) in the nuclear fraction from 19.5 ± 2.5% at 30 μM to 40 ± 2% at 60 μM in 4134/Late cells (Figure 1C) and from 18 ± 3% at 30 μM to 44.5 ± 3.5% at 60 μM in BL41 cells (Figure 1D). Notably, in both cellular models, the NF-κB pathway was active under maintenance conditions, showing high expressions of nuclear p65 at the basal level (Figure 1C,D), likely dependent on the CD40 signalling [61,62,63]. In addition, co-immunoprecipitation assays show that TERT and p-p65 were associated in complexes in 4134/Late cells under maintenance conditions (Figure S3) and that BIBR treatment reduced the levels of both p-p65 and TERT in the TERT/p-p65 complex (Figure S3).

### 3.2. TERT Inhibition by BIBR Suppressed Transcription of a Subset of NF-κB Target Genes, including MYC

As both the phosphorylation and nuclear localization of NF-κB p65 are important for its transcriptional function [64], the transcription levels of NF-κB target genes in LCL and BL cells following treatment with TERT inhibitor were analysed. BIBR treatment, even at a low concentration of 30 µM, caused a significant decrease in the transcription of NF-κB target genes *MYC*, *nuclear factor of kappa light polypeptide gene enhancer in B-cells inhibitor, alpha* (*IκBα)*, *BCL2 apoptosis regulator* (*BCL2)*, and *Survivin*, involved in cellular proliferation, DNA replication, and apoptosis in both 4134/Late (Figure S4A) and BL41 (Figure S4B) cells.

**Figure 2:**
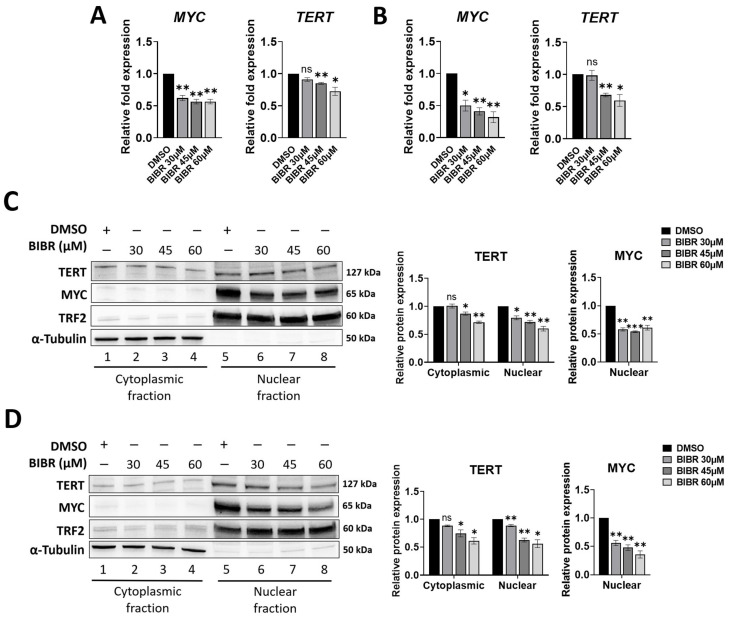
Telomerase reverse transcriptase (TERT) inhibition downregulated MYC proto-oncogene bHLH transcription factor (MYC) levels. (**A**,**B**) Cells were treated with BIBR at indicated concentrations or with DMSO as a control for 24 h. Levels of relative mRNA expression for *MYC* and *TERT* in 4134/Late (**A**) and BL41 (**B**) cells are shown. Data represent the mean and SD (bar) from three separate experiments. (**C**,**D**) Cells treated with BIBR at indicated concentrations or DMSO as a control for 24 h were processed to obtain cytoplasmic and nuclear extracts. Representative Western blots showing cytoplasmic and nuclear protein levels of TERT, MYC, TRF2, and α-tubulin in 4134/Late (**C**) and BL41 (**D**) cells are shown. α-tubulin and TRF2 were used as loading controls for the cytoplasmic and nuclear fractions, respectively. The original Western blots are shown in File S1. Graphs next to the blots show the values in arbitrary units of densitometric analysis performed with ImageJ software. Data represent the mean and SD (bar) from three separate experiments. A significant difference between values in BIBR-treated vs. DMSO-treated cells is shown: * *p* < 0.05; ** *p* < 0.01; *** *p* < 0.001; ns: not significant.

Given the pivotal role of MYC in EBV-driven B-cell proliferation and Burkitt’s lymphoma, its expression following treatment with TERT inhibition was analysed in more detail. Results show that the significant decrease in *MYC* mRNA level induced by BIBR treatment (Figure 2A,B) was paralleled by a concomitant decrease in its nuclear protein expression: from 42 ± 2% at 30 μM to 39 ± 3% at 60 μM (*p* < 0.01) in 4134/Late cells (Figure 2C) and from 44 ± 3% at 30 μM to 64.5 ± 4.5% at 60 μM (*p* < 0.01) in BL41 cells (Figure 2D).

BIBR treatment per se does not directly impact the *TERT* transcriptional level. Nonetheless, BIBR treatment decreases both NF-κB p65 and MYC nuclear levels, transcriptional factors of the *TERT* promoter [34,35,39,65]. In line with these observations, short-term BIBR treatment reduced *TERT* mRNA levels in both 4134/Late (Figure 2A) and BL41 (Figure 2B) cells. Consistently, TERT protein was also expressed at low levels in both 4134/Late (Figure 2C) and BL41 (Figure 2D) BIBR-treated cells.

### 3.3. MYC Deregulation Mediated by TERT Inhibition Was Independent of WNT/β-Catenin Signalling, and TERT and MYC Did Not Interact at the Protein Level

Besides NF-κB signalling, the *MYC* oncogene is transcribed by the WNT/β-catenin pathway [66]. TERT’s involvement in the regulation of WNT/β-catenin has been extensively documented [30,32,33,67,68]; thus, the possibility that *MYC* transcriptional downregulation following TERT inhibition could be mediated via WNT/β-catenin was investigated by evaluating the transcriptional levels of *β-catenin* (*CTNNB1*) and the WNT/β-catenin target genes *axin 2* (*AXIN2*) and *cyclin D1* (*CCND1*) following BIBR treatment. Results show that there was no significant change in the mRNA levels of *CTNNB1* and *AXIN2* in both 4134/Late (Figure S5A) and BL41 (Figure S5B) BIBR-treated cells compared to controls. Similarly, the small increase in *CCND1* transcription observed in 4134/Late cells (Figure S5A) was not significant. As expected, the expression of *CCND1* in BL41 cells was not found, in agreement with the data that most B-cell lymphomas do not express cyclin D1 [69].

It has been suggested that TERT functions as a cofactor in MYC-dependent transcription by binding MYC protein and consequently improving MYC stability and accessibility in its target promoters [29,30]. To assess this possibility in our cellular models, the association between MYC and TERT proteins was checked through a co-immunoprecipitation assay. As shown in Figure S5, no binding interaction was found between endogenous MYC and TERT proteins in 4134/Late cells (Figure S5C) or BL41 cells (Figure S5D).

### 3.4. Ectopic TERT Expression Activated Transcription of NF-κB Target Genes

**Figure 3:**
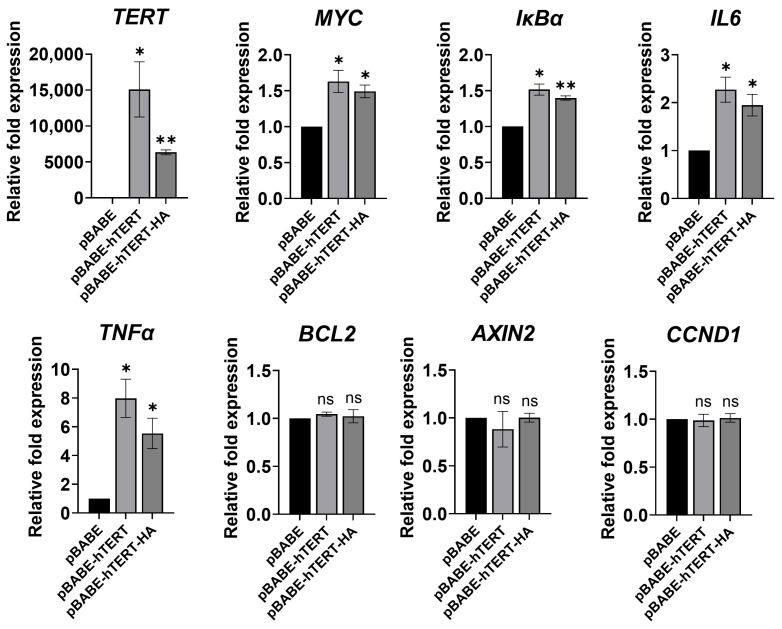
Ectopic expression of TERT or TERT-HA increased transcription of NF-κB target genes. U2OS cells were transfected with pBABE-hTERT, pBABE-hTERT-HA, or pBABE (control) vectors. Forty-eight h after transfection, RNA was harvested and mRNA levels for the genes indicated were determined by quantitative RT-PCR. Data represent the mean and SD (bar) from three separate experiments. A significant difference between values in pBABE-hTERT or pBABE-hTERT-HA transfected cells vs. control pBABE-transfected cells is shown: * *p* < 0.05; ** *p* < 0.01; ns: not significant.

To further elucidate the role of TERT in the NF-κB transcriptional program, the effects of ectopic TERT expression on NF-κB and WNT/β-catenin target genes were examined in U2OS cells. U2OS cells lack endogenous TERT expression with no detectable telomerase activity and maintain telomere length through the Alternative Lengthening of Telomeres mechanism [70]. *TERT* transfection efficiently increased its expression in the U2OS cell line (Figure 3), and ectopic TERT expression was accompanied by a significant increase in transcription of a subset of NF-κB target genes (Figure 3) but did not induce any change in transcription of the known WNT/β-catenin target genes *CCND1* [71] and *AXIN2* [72] (Figure 3).

To analyse whether the selective link between TERT and the NF-κB dependent transcriptional program was unrelated to the telomere maintenance mechanism, U2OS cells were also transfected with pBABE-puro-hTERT-HA, a plasmid expressing a derivative of the TERT protein that had been modified through the attachment of an HA epitope tag to its C terminus (hTERT-HA). hTERT-HA retains telomerase activity but lacks the ability to maintain telomere length [73,74]. Interestingly, as observed with the wild-type TERT (pBABE-hTERT), the ectopic expression of hTERT-HA also significantly increased the expression of the NF-κB target genes *MYC*, *IkBα*, *interleukin 6* (*IL6*), and *tumour necrosis factor* (*TNFα*) (Figure 3) without any change in transcription of WNT/β-catenin target genes *CCND1* and *AXIN2*. These results further indicate that TERT might have other cellular functions, e.g., modulation of NF-κB target genes, unrelated to its activity on telomeres.

### 3.5. p65 Inhibition Recapitulated the Effects of TERT Inhibition

**Figure 4:**
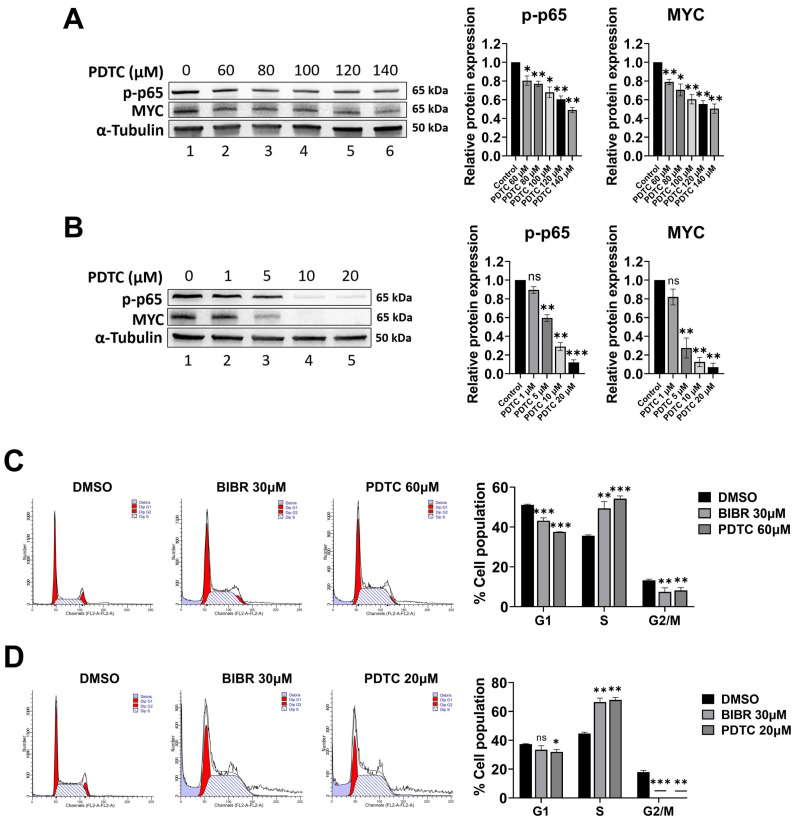
p65 inhibition recapitulated the effects of TERT inhibition on MYC and the cell cycle. (**A**,**B**) Representative Western blots showing total protein levels of p-p65 and MYC in 4134/Late (**A**) and BL41 (**B**) cells upon treatment with Ammonium pyrrolidine dithiocarbamate (PDTC) at the indicated concentrations for 24 h. α-tubulin was used as a loading control. The original Western blots are shown in File S1. Graphs on the right show the values in arbitrary units of densitometric analysis performed with ImageJ software. Data represent the mean and SD (bar) from two separate experiments. (**C**,**D**) 4134L/Late (**C**) and BL41 (**D**) cells were treated with 30 μM BIBR, PDTC at indicated concentrations, or DMSO as a control for 24 h, labelled with propidium iodide (PI) and analysed by flow cytometry for cell cycle profiles. Panels from a representative experiment are shown. Graphs on the right show percentages of cells in G1, S, and G2/M-phases, respectively. Values show the means and SD (bar) of three separate experiments. A significant difference between values in BIBR-treated or PDTC-treated cells vs. DMSO-treated cells is shown: * *p* < 0.05; ** *p* < 0.01; *** *p* < 0.001.

To determine the role of NF-κB p65 on MYC regulation in our in vitro models, the effects of the selective NF-κB p65 activity inhibitor (PDTC) [75] were evaluated on MYC expression. PDTC is a well-known NF-κB p65 inhibitor that efficiently reduces p-p65 accumulation [76,77]. As expected, PDTC, in a dose-dependent manner, reduced the p-p65 levels in both 4134/Late (Figure 4A) and BL41 (Figure 4B) cells. NF-κB signalling inhibition by PDTC decreased MYC protein expression in both 4134/Late (Figure 4A) and BL41 (Figure 4B) cells in a dose-dependent manner. Interestingly, both BIBR and PDTC treatment altered the cell cycle profile in 4134/Late (Figure 4C) and BL41 (Figure 4D) cells, with a significant accumulation of cells in the S-phase (*p* < 0.01 for both cell lines).

### 3.6. TERT Inhibition Promoted P21 Expression and Nuclear Localization

**Figure 5:**
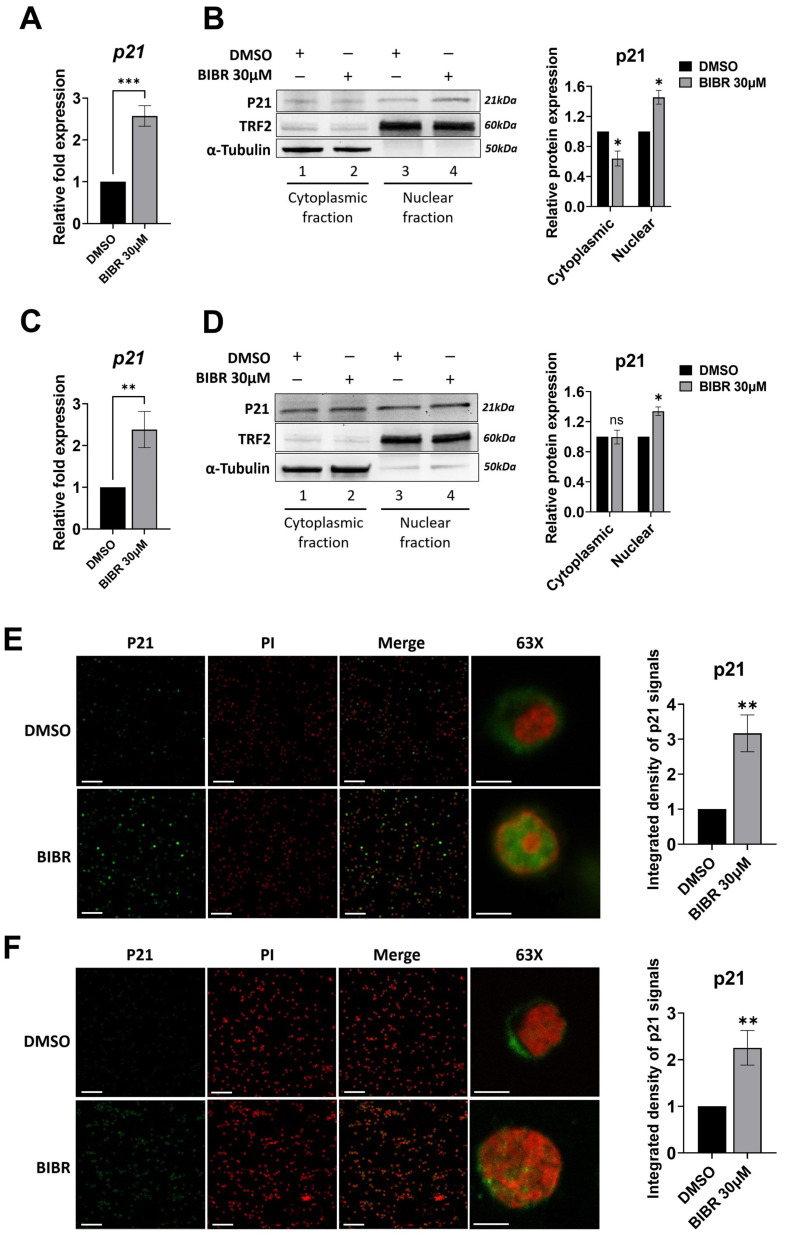
Telomerase inhibition enhanced the expression and nuclear localization of cyclin-dependent kinase inhibitor 1A (CDKN1A, P21). (**A**,**C**) Cells were treated with 30 μM BIBR or DMSO as a control for 24 h. Levels of relative mRNA expression for *P21* in 4134/Late (**A**) and BL41 (**C**) are shown. Data represent the mean and SD (bar) from three separate experiments. (**B**,**D**) 4134/Late (**B**) and BL41 (**D**) cells treated with 30 μM BIBR or DMSO as a control for 24 h were processed to obtain cytoplasmic and nuclear extracts. The indicated proteins were probed by Western blotting and representative blots are shown. α-tubulin and TRF2 were used as loading controls for the cytoplasmic and nuclear fractions, respectively. The original Western blots are shown in File S1. Graphs show the values in arbitrary units of densitometric analysis performed with ImageJ software. Data represent the mean and SD (bar) from three separate experiments. (**E**,**F**) Representative immunofluorescence images showing P21 (green), PI (nuclear marker, red), and their merge in 4134/Late (**E**) and BL41 (**F**) cells treated with 30 μM BIBR or DMSO as a control (magnification 20× unless specified). Graphs show the values in arbitrary units of densitometric analysis performed with ImageJ software. Data represent the mean and SD (bar) from two separated experiments, scale bars: 100 μm in 20× and 5 μm in 63× images. A significant difference between values in BIBR-treated vs. DMSO-treated cells is shown: * *p* < 0.05; ** *p* < 0.01; *** *p* < 0.001; ns: not significant.

P21 is an important cell-cycle inhibitor [78]. As MYC is a repressor of *P21* transcription [78] and MYC is downregulated in BIBR-treated cells, the effects of short-term TERT inhibition on P21 expression were investigated. The results show that the S-phase cell cycle arrest induced by TERT inhibition was characterized by a significant increase in *P21* mRNA expression in both 4134/Late (*p* < 0.001, Figure 5A) and BL41 (*p* < 0.01 Figure 5C) cells. As shown in Figure 5, a significantly increased P21 nuclear accumulation was observed in both 4134/Late (Figure 5B) and BL41 (Figure 5D) cells. Consistently, P21 expression and accumulation in the nuclear compartment were further confirmed by immunofluorescence in 4134/Late (Figure 5E) and BL41 (Figure 5F) BIBR-treated cells. This is of interest, given that P21 nuclear accumulation is associated with cell cycle inhibitory functions, whereas its cytoplasmic localization is often associated with pro-oncogenic activities [78,79].

### 3.7. Short-Term Tert Inhibition by BIBR in Zebrafish Reduced Myc and Increased p21 Expression

**Figure 6:**
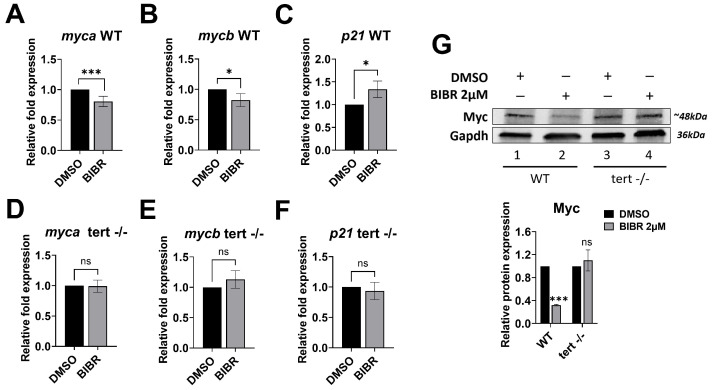
BIBR treatment downregulated the expression of zebrafish *myca* and *mycb* and upregulated *p21*. Twelve hours post fertilization (hpf), zebrafish wild-type (WT) and *tert* mutant (*tert^hu3430/hu3430^; tert −/−)* embryos were treated with 2 μM BIBR or DMSO as a control for 12 h, from 12 to 24 hpf. Levels of relative mRNA expression for the indicated genes in WT (**A**–**C**) and *tert* mutant (**D**–**F**) embryos are shown. Data represent the mean and SD (bar) from three separate experiments. (**G**) Representative Western blots showing total protein levels of Myc in WT and *tert* mutant zebrafish embryos upon treatment with 2 μM BIBR for 12 h. Glyceraldehyde-3-phosphate dehydrogenase (Gapdh) was used as a loading control. The original Western blots are shown in File S1. The graph shows the values in arbitrary units of densitometric analysis performed with ImageJ software. Data represent the mean and SD (bar) from two separate experiments. A significant difference between values in BIBR-treated embryos vs. DMSO-treated embryos is shown: * *p* < 0.05; *** *p* < 0.001; ns: not significant.

Previously, we used the zebrafish model to confirm in vivo our in vitro data on the telomere length-independent anti-proliferative effect of telomerase inhibition [21]. Therefore, we evaluated whether the modulatory role played by Tert during cell cycle progression could be associated with the variation in *myc* and *p21* expression in the zebrafish model as observed in vitro. As shown in Figure 6, in WT zebrafish embryos, 12 h of treatment with 2 µM BIBR, from 12 to 24 hpf, induced a modest but significant decrease of both zebrafish *MYC* orthologs [80] *myca* (19.3 ± 8.4%, *p* < 0.001) (Figure 6A) and *mycb* (17.7 ± 10.9%, *p* = 0.017) (Figure 6B) expression compared to the controls. Conversely, BIBR treatment shows no effect on *myca* (Figure 6D) or *mycb* (Figure 6E) expression in *tert−/−* embryos. Consistently, Myc protein was also decreased in BIBR-treated WT embryos but not in *tert−/−* ones (Figure 6G).

Similar to the in vitro results, 12 h of treatment with 2 µM BIBR shows a significantly increased *p21* expression compared to the DMSO-treated controls in WT (33.8 ± 18.2%; *p* = 0.01) but not in *tert−/−* embryos (Figure 6C and Figure 6F, respectively).

### 3.8. Anti-Proliferative Effects of Combined Treatment with BIBR and FLU or CY in EBV-Immortalized and Fully Transformed B Cells Xenografted in Zebrafish

The previous in vitro observation that TERT inhibition by BIBR in combination with FLU or CY (two of the agents most frequently used to treat B-cell malignancies) showed a significant alteration of cell growth with respect to treatment with chemotherapeutic agents alone [12] prompted us to investigate whether TERT inhibition also increased susceptibility to antineoplastic drugs in an in vivo context. To this end, labelled EBV-immortalized and fully transformed B cells were treated or untreated with BIBR, xenografted in casper zebrafish embryos, and subsequently exposed or unexposed to chemotherapeutic agents. The number of injected cells was monitored by flow cytometry analysis in short-term experiments to avoid the expected telomere shortening influence on proliferation due to the inhibition of canonical TERT activity on telomeres widely demonstrated under long-term BIBR treatment [81,82].

**Figure 7:**
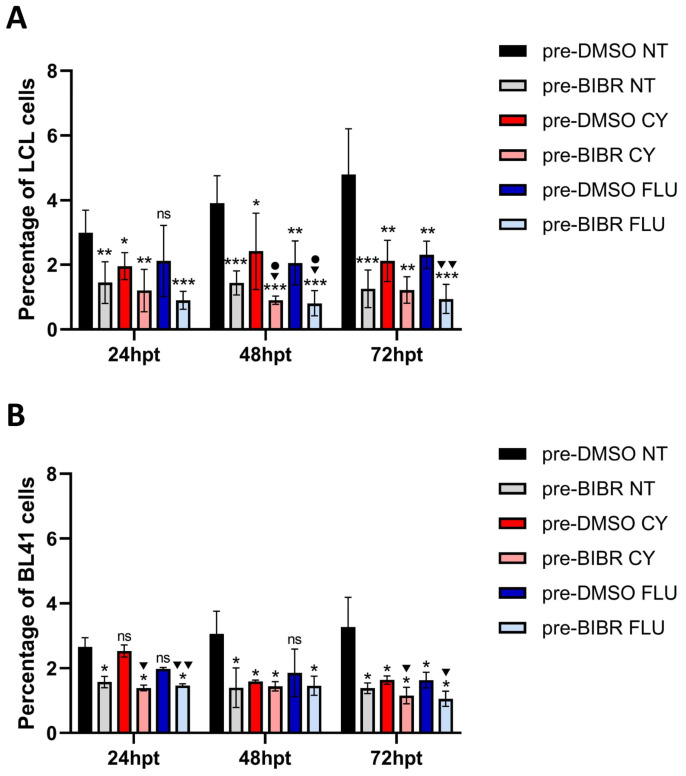
Combined treatment with BIBR and fludarabine (FLU) or cyclophosphamide (CY) inhibited the growth of EBV-immortalized and fully transformed B cells xenografted in zebrafish. 4134/Late (**A**) and BL41 (**B**) cells were pre-treated with 30 μM BIBR (pre-BIBR) or DMSO (pre-DMSO) as a control for 16 h, stained with CM-DiI, and xenotransplanted in the yolk sac of 72 hpf embryos. Twenty-four hours post-xenotransplantation, xenografted embryos were divided into groups untreated (NT) or treated with FLU or CY. The percentage of tumour cells in engrafted embryos was determined at 24, 48, and 72 h post-treatment (hpt) by flow cytometric analysis. Data represent the mean and SD (bar) from three separate experiments with 10 embryos per group. ns: not significant, *, ^▼^ and • *p* < 0.05; ** and ^▼▼^ *p* < 0.01; *** *p* < 0.001, where (*) asterisks represent the statistical significance vs. the pre-DMSO NT group; the (▼) symbol shows the statistical significance of the combined treatment with BIBR and CY or FLU vs. the respective drug treatment alone and the (•) symbol shows the statistical significance of the combined treatment with BIBR and CY or FLU vs. the BIBR treatment alone.

Results show that xenografted untreated 4134/Late cells (pre-DMSO NT) proliferated in zebrafish embryos throughout the experimental period (Figure 7A). In comparison, BIBR (pre-BIBR NT) and drug treatment (pre-DMSO CY and pre-DMSO FLU) decreased xenografted 4134/Late cells proliferation rate compared to control cells in untreated embryos (pre-DMSO NT), with BIBR (pre-BIBR NT) showing a higher inhibitory effect at each time point (Figure 7A). The combined treatment of BIBR and each drug (pre-BIBR CY and pre-BIBR FLU) further impaired xenografted cells’ proliferation rate compared to the control cells in untreated embryos (pre-DMSO NT) at each considered time point (Figure 7A). Notably, the combined treatment of BIBR and CY significantly reduced xenografted LCL cells’ proliferation rate, also compared to those observed with the drug treatment alone at 48 hpt (1 ± 0.25% vs. 2.41 ± 1.18%, *p* = 0.031, percentage of engrafted cells in pre-BIBR CY vs. pre-DMSO CY). This cumulative anti-proliferative effect was even more evident with the combined treatment with BIBR and FLU being significant both at 48 hpt (0.81 ± 0.39% vs. 2.05 ± 0.68%, *p* = 0.014, in pre-BIBR FLU vs. pre-DMSO FLU) and 72 hpt (0.94 ± 0.45% vs. 2.3 ± 0.42%, *p* = 0.002, in pre-BIBR FLU vs. pre-DMSO FLU) (Figure 7A). Parallel experiments conducted by xenografting BL41 cells show similar results, with a significant anti-proliferative effect of the combined treatments at 72 hpt (1.16 ± 0.26% vs. 1.63 ± 0.13%, *p* = 0.044, in pre-BIBR CY vs. pre-DMSO CY and 1.06 ± 0.23% vs. 1.63 ± 0.25%, *p* = 0.025, in pre-BIBR FLU vs. pre-DMSO FLU) (Figure 7B). These results reflect our previous in vitro data [12] and indicate that TERT inhibition by BIBR enhanced the anti-proliferative effects of chemotherapeutic agents.

## 4. Discussion

Telomerase, given its telomeric function that provides unlimited replicative potential, plays a critical role in tumour formation and progression. Nevertheless, many studies have highlighted the importance of this enzyme in several other pro-tumourigenic processes, independently of telomere maintenance [23,24,29,30,32,33,83,84]. However, the molecular mechanism(s) by which telomerase may contribute to oncogenesis beyond telomere maintenance have not been fully clarified and probably depend on the cellular context.

Here we show that in in vitro models of B-cell lymphoproliferative disorders, i.e., LCL, and B-cell malignancies, i.e., BL, TERT inhibition impairs the transcription of a subset of NF-κB target genes, i.e., *MYC*, *IκBα*, *BCL2*, and *Survivin*. NF-κB is one of the well-known transcriptional regulators of *TERT* either directly binding to the *TERT* promoter [34] and/or indirectly through transcriptional activation of *MYC* [35]. We show a link between TERT and NF-κB p65, whereby TERT, through extra-telomeric function, regulates nuclear levels of p65, a phenomenon that promotes the NF-κB signalling pathway. The evidence that only the nuclear protein levels of p65 are affected by TERT inhibition, without any change in mRNA expression, suggests that TERT favours the stability of nuclear DNA-bound p65 by reducing its ubiquitination and proteasomal degradation, which are known to constitute downstream events playing major roles in limiting the intensity and duration of NF-κB p65 activity [85]. On this basis, we show that TERT forms a complex with p-p65 under maintenance conditions, thereby putting forward an extra-telomeric function of TERT in NF-κB p65 signalling that can be targeted by BIBR. Interestingly, Ghosh and colleagues demonstrated that the TERT inhibitor MST-312, which, similar to BIBR, disturbs the TEN domain conformation of TERT [16], reduced levels of p65 occupancy at NF-κB target sites [23]. Thus, it is conceivable that BIBR, similarly to MST-312, alters the TERT/p65 complex at NF-κB target genes, consequently affecting their transcriptional expression. Indeed, our results demonstrate that TERT inhibition by BIBR leads to a decrease in nuclear p65 levels, thus altering the transcription of a subset of NF-κB targets, including *MYC*. The finding that the NF-κB p65 inhibitor, i.e., PDTC, led to a decrease in MYC protein level in both 4134/Late and BL41 cells and an altered cell cycle profile in these cellular models, leading to an S-phase cell cycle arrest, supports the idea that the cell cycle arrest induced by TERT inhibition in vitro [12] occurs through mechanism(s) involving NF-κB and MYC. Notably, 4134/Late cells were found to be more resistant to NF-κB p65 inhibition compared to BL41 cells, an effect that is likely due to EBV infection, as the viral latent membrane protein 1 (LMP1) regulates NF-κB p65 activation [86] and nuclear translocation [87].

The involvement of TERT in NF-κB signalling has been further sustained by the results from experiments with ectopic TERT expression, which increased transcription of several NF-κB p65 target genes in a telomerase-negative cell line. Finding that a biologically inactive TERT overexpression (hTERT-HA) also increased NF-κB target genes transcription in telomerase-negative cells further sustains that TERT has a non-canonical function in modulating NF-κB signalling regardless of its ability to promote telomere lengthening. Notably, the link between TERT and the NF-κB pathway we observed in LCL cells is in agreement with our previous observation in the context of the crosstalk between EBV and telomerase, as we demonstrated that in LCLs, TERT induces *notch receptor 2* (*NOTCH2)* expression through NF-κB signalling, and NOTCH2, in turn, through basic leucine zipper ATF-like transcription factor (BATF) expression, represses BamHI Z fragment leftward open reading frame 1 (BZLF1), the master regulator of the EBV lytic cycle [51,88].

TERT has been reported to be involved in the transcriptional regulation of WNT target genes, including *MYC*, through its interaction with the chromatin regulator BRG1 [28]. We did not observe transcriptional downregulation of known WNT target genes *AXIN2* and *CCND1* after TERT inhibition by BIBR treatment in our in vitro models. Furthermore, we show that ectopic over-expression of TERT did not alter the transcription of WNT target genes, further confirming that under our experimental conditions, TERT was not involved in WNT signalling regulation. Our results agree with those of Ghosh et al. [23], who, following TNFα stimulation, observed no association of TERT with BRG1 but reported that the telomerase inhibition significantly limits TNFα-mediated p65 binding to a subset of NF-κB dependent promoters [23].

We also found that cell cycle arrest induced by TERT inhibition is characterized by increased levels of P21, a well-known cell cycle inhibitor. As MYC is a down-regulator of P21 [89,90], the increase of this protein may be linked to the downregulation of MYC induced by TERT inhibition. In the nucleus, P21 exerts its cell cycle inhibitory function by interfering with proliferating cell nuclear antigen (PCNA)-dependent DNA polymerase activity and/or inhibiting cyclin dependent kinase 2 (CDK2)-dependent replication origin firing, ultimately inhibiting DNA replication, and prolonging the S-phase [78]. S-phase lengthening indicates replication fork stalling, which activates DDR, which is crucial for fork protection [91]. These results provide a conceivable explanation of how telomerase inhibition can lead to cell cycle arrest with the activation of telomere length-independent DDR that we previously observed in in vitro models [12].

Importantly, using the zebrafish model, we confirm that also in the in vivo system, Tert inhibition is associated with downregulation of Myc and increased expression of *p21*, thus accounting for the impaired proliferation and cell cycle arrest with the activation of telomere length-independent DDR, which we have previously observed upon Tert inhibition in this animal model [21]. Notably, these effects were specifically related to Tert inhibition since BIBR treatment shows no effect on Myc and *p21* expression in *tert−/−* embryos. The lack of difference in Myc expression between untreated *tert−/−* and WT embryos suggests that Tert function(s) is partially compensated in zebrafish *tert−/−*. While alternative pathways might compensate for the non-canonical functions of telomerase in *tert−/−* embryos, as suggested in the context of Tert−/− mice [33], the acute inhibition of Tert in WT embryos causes the significant effects we observed in our experiments. Altogether, these findings enforce the concept that telomerase per se seems to exert growth-promoting activities that are independent of its canonical role in telomere length maintenance and sustain the interest in TERT inhibition as an anticancer strategy.

The evidence that TERT inhibition in combination with both CY and FLU shows a cumulative inhibitory effect on the proliferation of both EBV immortalized and fully transformed B cells xenografted in vivo compared to the single agent treatment strongly sustains the validity of this strategy to counteract tumour growth. This combined strategy, which rapidly impairs tumour cell proliferation and sensitizes cancer cells to cytotoxic effects of chemotherapeutic agents, could achieve superior therapeutic outcomes compared to current treatment modalities. Targeting TERT for cancer treatment is not a novel concept given the specificity of TERT expression in tumour cells for the maintenance of telomere length and the replicative potential. However, strategies selectively inducing shortening or damaging telomeres may be limited by normal tissue toxicity inherent to the long-term treatment required before anti-tumour effects caused by critical telomere attrition are exerted, as observed in clinical trials with Imetelstat, an oligonucleotide complementary to the TERC template region that competitively inhibits telomerase activity at telomeres [92,93]. Despite the lack of efficacy in targeting telomere maintenance and concern about side effects of long-term telomerase inhibition, transiently inhibiting TERT non-canonical function(s) to impact tumour growth and survival may offer an opportunity for tumour-specific sensitization to therapy, as we observed in the present study.

Under normal conditions, somatic cells have no detectable telomerase activity, but in cancer cells, TERT is reactivated and can contribute to oncogenesis by establishing a feed-forward signalling loop with NF-κB signalling: *TERT* is transcriptionally upregulated by NF-κB either directly and/or through MYC; in turn, TERT facilitates p65 in its transcriptional program by enhancing p65 nuclear levels, thereby leading to enhanced expression of NF-κB p65 target genes, including MYC, which is a repressor of P21, a pivotal inhibitor of the cell cycle. This leads to the formation of a multifaceted regulatory loop between TERT, NF-κB p65, and MYC. This regulatory loop, once activated, can contribute to tumour progression through the regulation of multiple hallmarks of cancer.

A schematic model summarizing the mechanism of TERT inhibition-mediated cell cycle arrest in EBV-immortalized and fully transformed B cells is in Supplementary Figure S6. The TERT inhibition by BIBR downregulates NF-κB p65 nuclear localization, reducing the availability of p65 on its target promoters and thereby decreasing the transcription of a subset of NF-κB p65 target genes, including *MYC*, *IκBα*, *BCL2*, and *Survivin*. The decreased NF-κB p65 and MYC protein levels compromise *TERT* promoter activation, reducing TERT expression. Furthermore, MYC downregulation compromises cellular proliferation by upregulating P21 expression and its nuclear localization, thereby leading to cell cycle arrest, which may ultimately contribute to the activation of DDR.

## 5. Conclusions

In conclusion, our results provide insight into the interdependency of various tumour-promoting factors and how short-term targeting of TERT can contribute to therapeutic effects beyond telomere maintenance. These results strongly support the evidence that TERT has telomere length-independent non-canonical functions in NF-κB p65 signalling and that telomerase inhibition can directly inhibit the transcription of NF-κB target genes, including MYC. Although this study implies the involvement of NF-κB and MYC as mediators of TERT extra-telomeric functions, other mechanism(s) and signals could be relevant in different cellular contexts. Furthermore, TERT inhibition in combination with both CY or FLU shows a cumulative inhibitory effect on the proliferation of EBV immortalized and fully transformed B cells in vivo, thus sustaining the concept that targeting TERT can be exploited as an efficient anticancer approach to enhance the therapeutic benefits of existing chemotherapeutic protocols regardless of telomere length and erosion. Given the potential therapeutic impact of these results, further studies with other specific TERT/telomerase inhibiting strategies and using patient-derived xenograft models should be undertaken to extend and validate these findings.

## Data Availability

The data presented in this study are available within the article or in the Supplementary Materials.

## Supplementary Materials

The following supporting information can be downloaded at: https://www.mdpi.com/article/10.3390/cancers15102673/s1, File S1: Full-length blots; File S2: Supplementary Table and Figures: Table S1: q-PCR primers for gene expression analysis; Figure S1: Effect of treatment with different doses of drugs on zebrafish embryo viability; Figure S2: Effects of different doses of BIBR on proliferation rate and telomere length in LCL and BL cells; Figure S3: BIBR treatment reduced the levels of TERT/p-p65 complexes; Figure S4: BIBR treatment downregulated the expression of a subset of NF-κB target genes; Figure S5: MYC downregulation in BIBR-treated cells is independent of WNT/β-catenin signalling; Figure S6: Schematic graph of the consequences of short-term TERT inhibition in EBV-immortalized and fully transformed B cells.
