# Supplementary material for: Short-Term TERT Inhibition Impairs Cellular Proliferation via a Telomere Length-Independent Mechanism and Can Be Exploited as a Potential Anticancer Approach
Source: Cancers (Basel). 2023 May 9;15(10):2673. doi: 10.3390/cancers15102673 (PMC10216832; doi:10.3390/cancers15102673)

Figure 1

Fig. 1C
p65 65 kDa

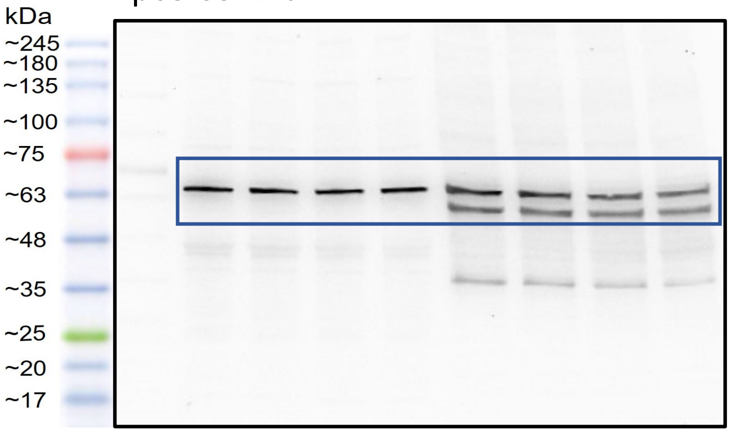

Fig. 1C
p-p65 65 kDa

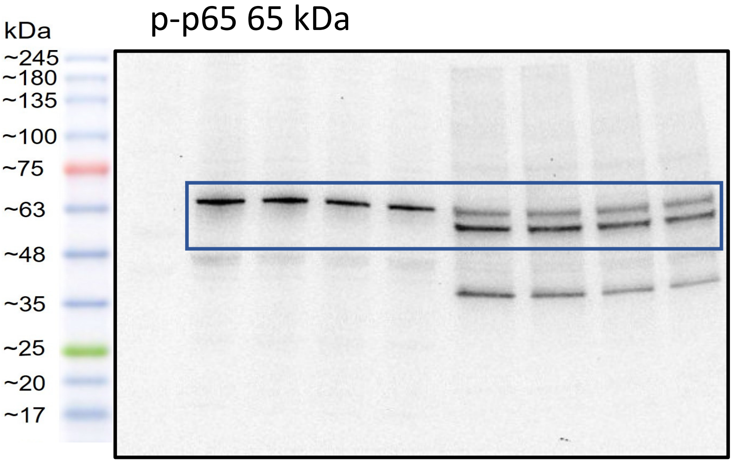

Fig. 1C
TRF2 60 kDa

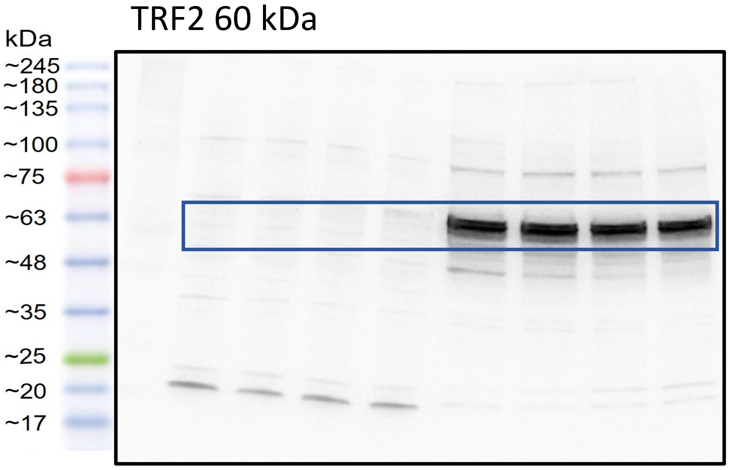

Fig. 1C
α-Tubulin 50 kDa

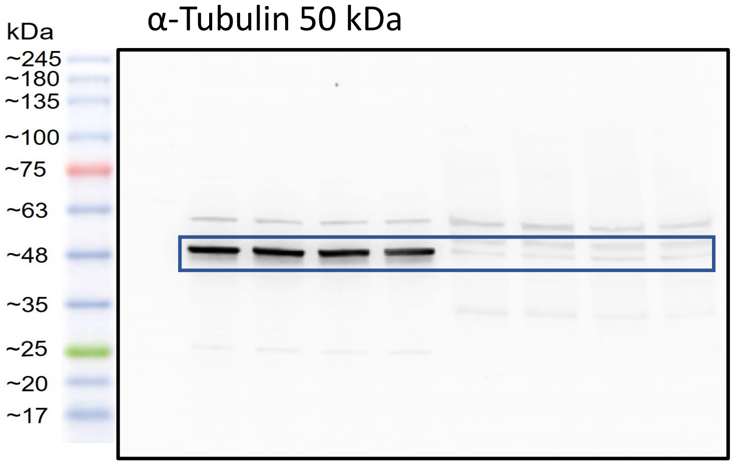

Fig. 1D
p65 65 kDa

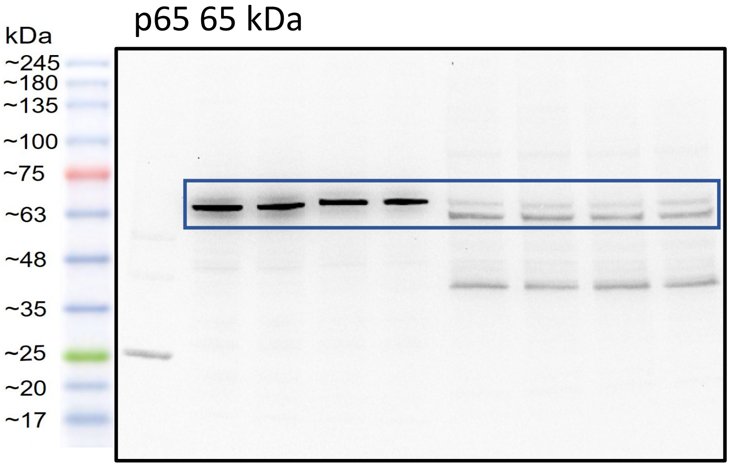

Fig. 1D
p-p65 65 kDa

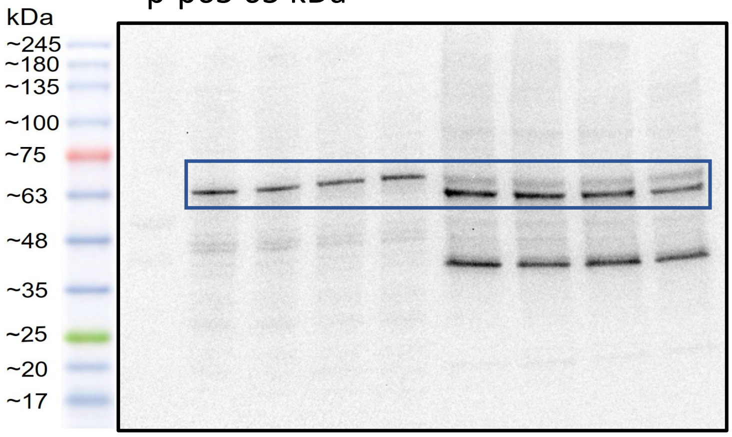

Fig. 1D
TRF2 60 kDa

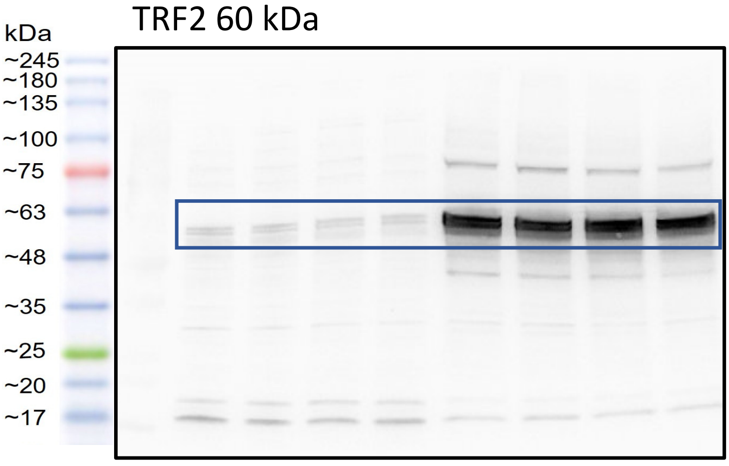

Fig. 1D
α-Tubulin 50 kDa

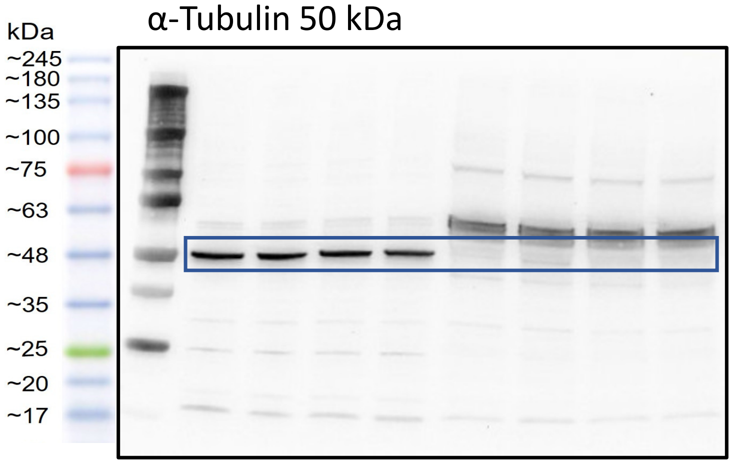

Figure 2

Fig. 2C
TERT 127 kDa

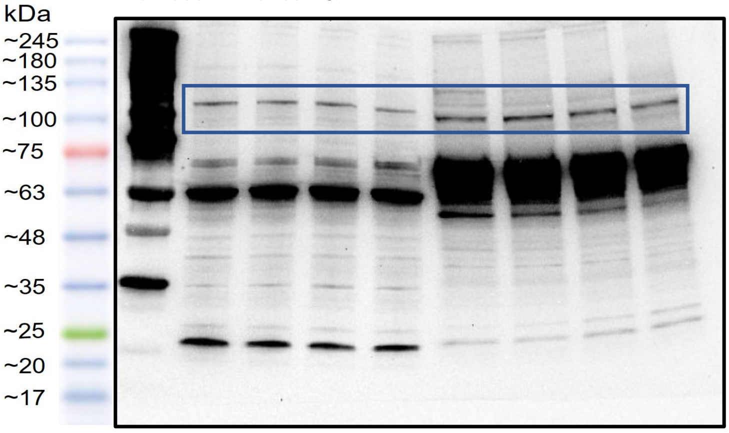

Fig. 2C
MYC 65 kDa

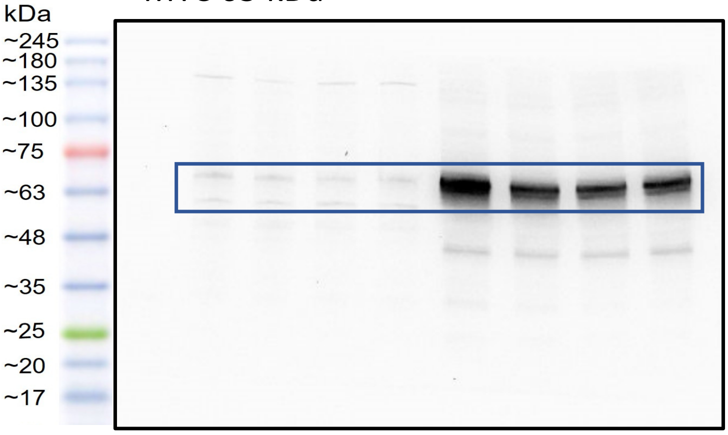

Fig. 2C
TRF2 60 kDa

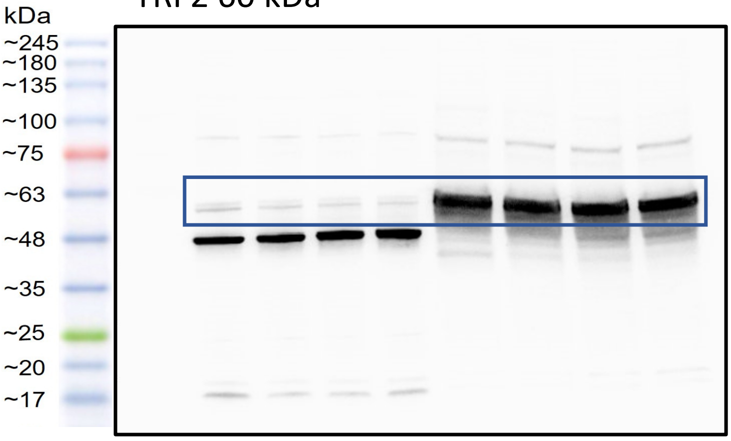

Fig. 2C
α-Tubulin 50 kDa

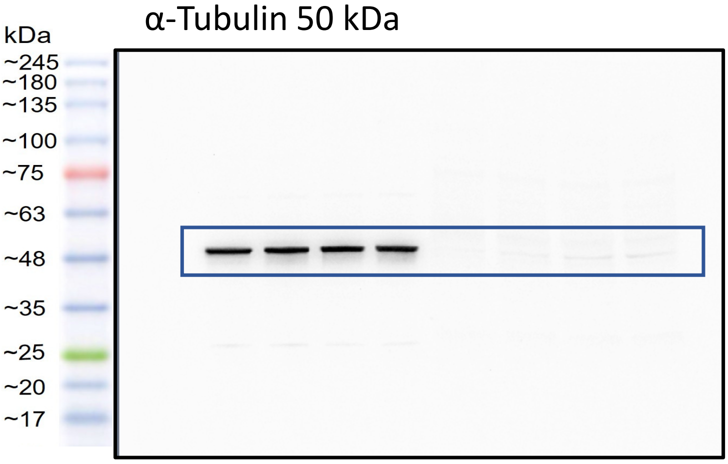

Fig. 2D
TERT 127 kDa

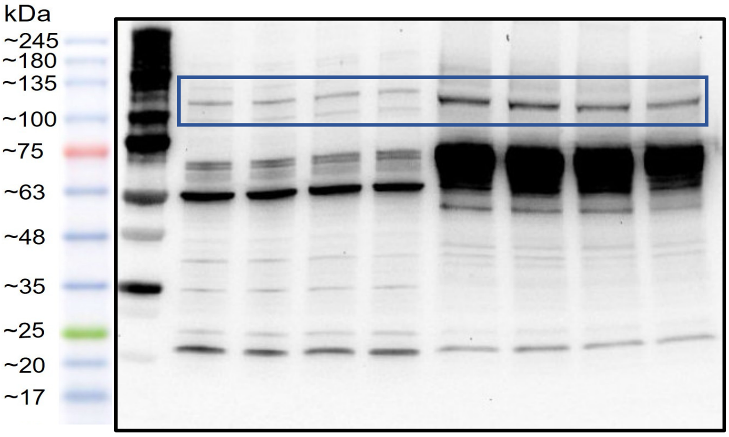

Fig. 2D
MYC 65 kDa

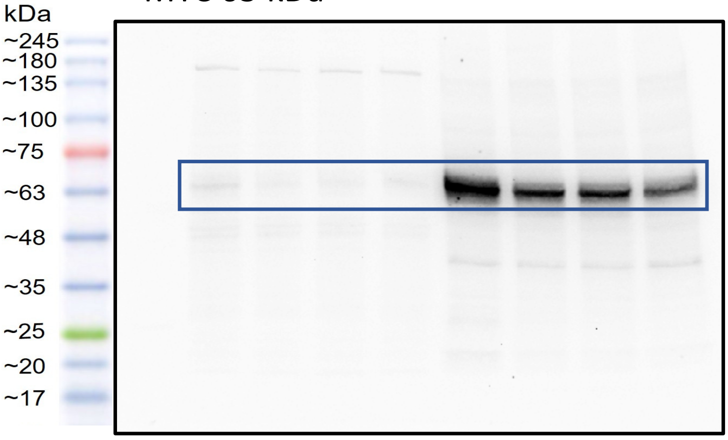

Fig. 2D
TRF2 60 kDa

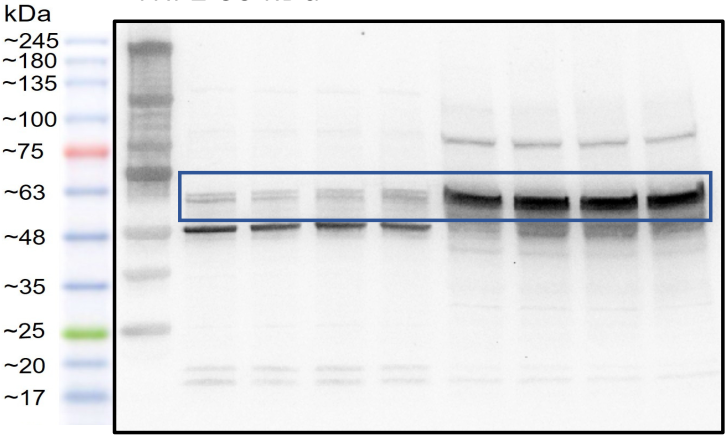

Fig. 2D
α-Tubulin 50 kDa

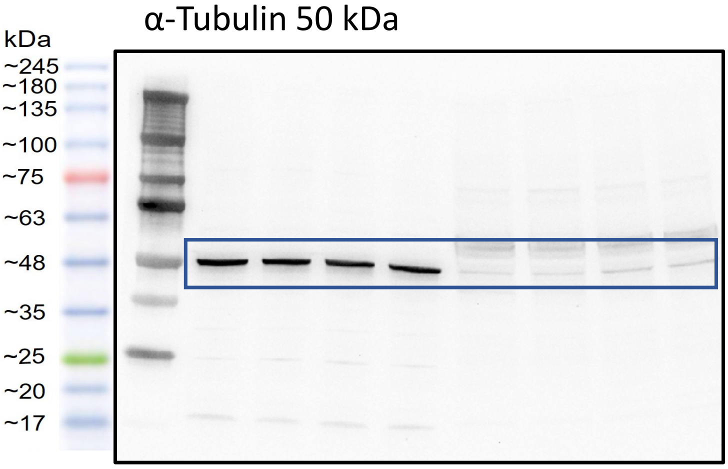

Figure 4

Fig. 4A
p-p65 65 kDa

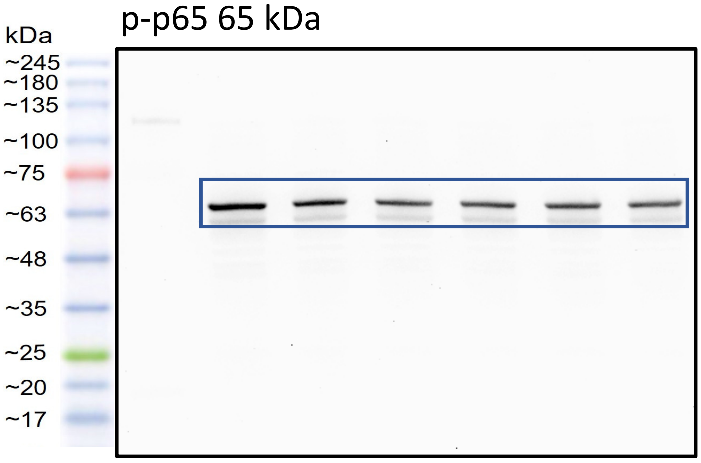

Fig. 4A
MYC 65 kDa

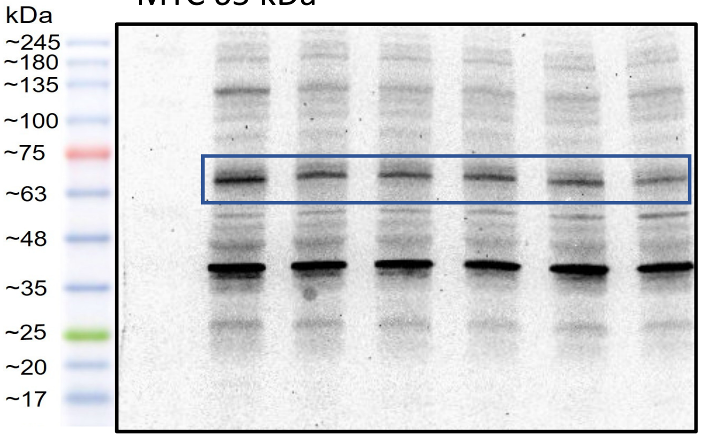

Fig. 4A
α-Tubulin 50 kDa

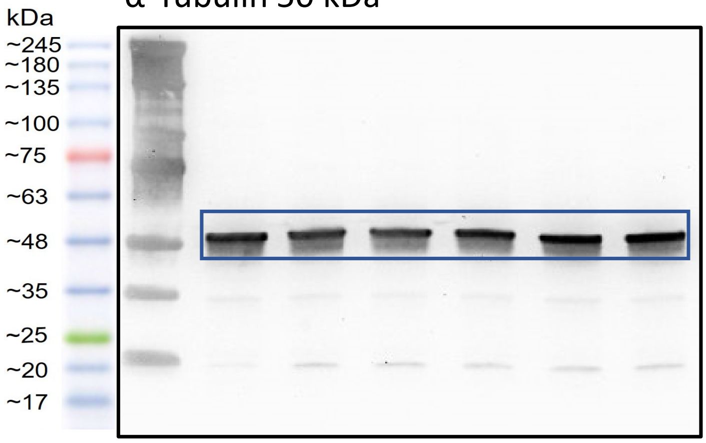

Fig. 4B
p-p65 65 kDa

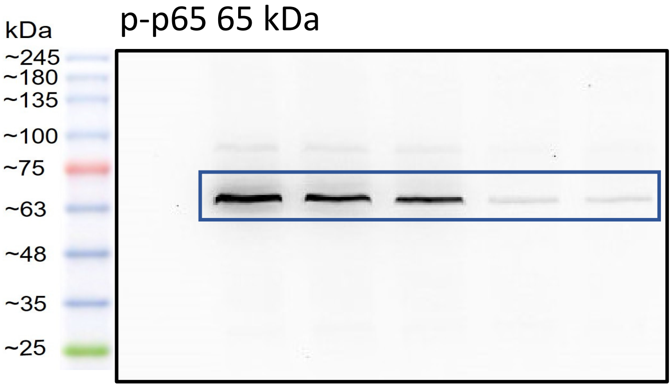

Fig. 4B
MYC 65 kDa

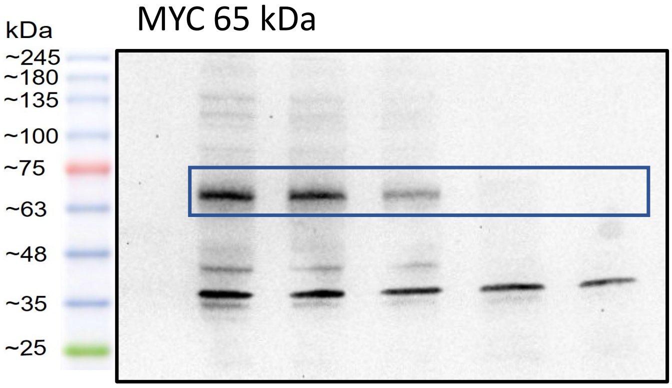

Fig. 4B
α-Tubulin 50 kDa

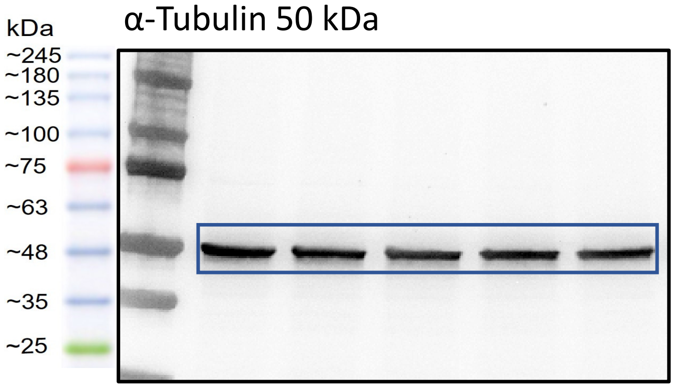

Figure 5

Fig. 5B
P21 21 kDa

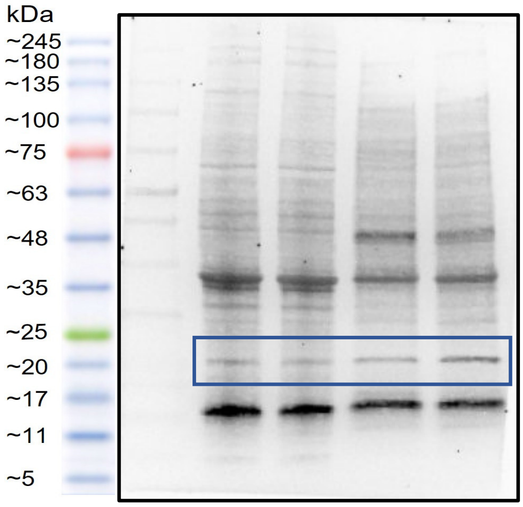

Fig. 5B
TRF2 60 kDa

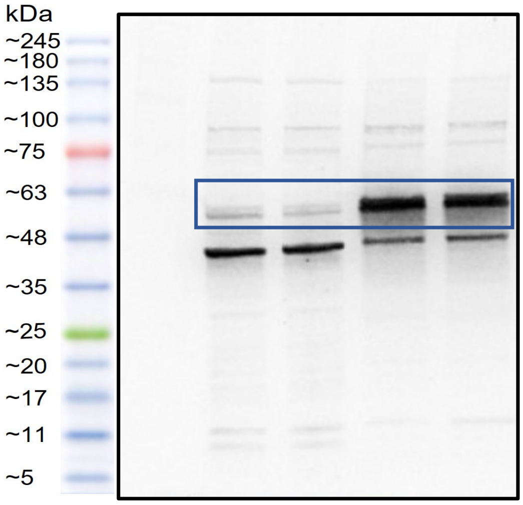

Fig. 5B
α-Tubulin 50 kDa

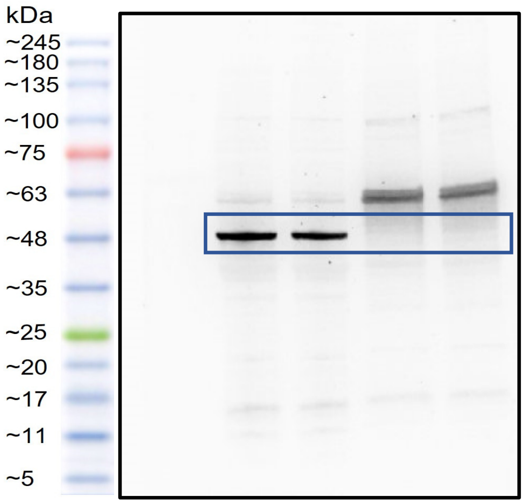

Fig. 5D
P21 21 kDa

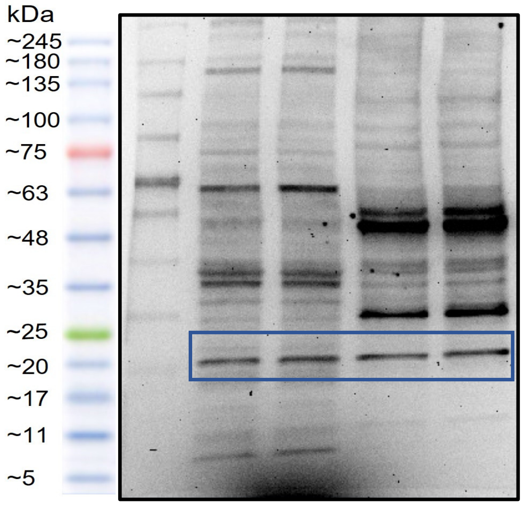

Fig. 5D
TRF2 60 kDa

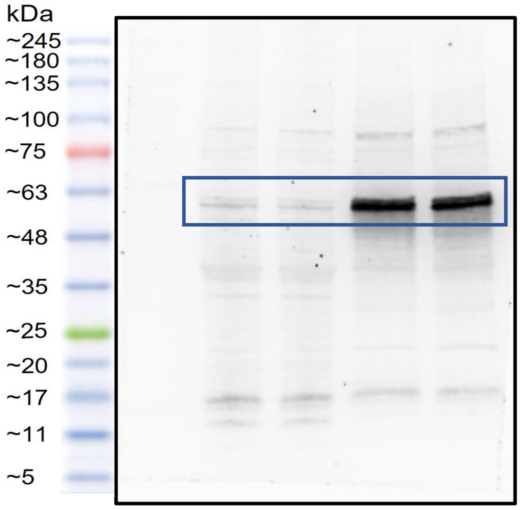

Fig. 5D
α-Tubulin 50 kDa

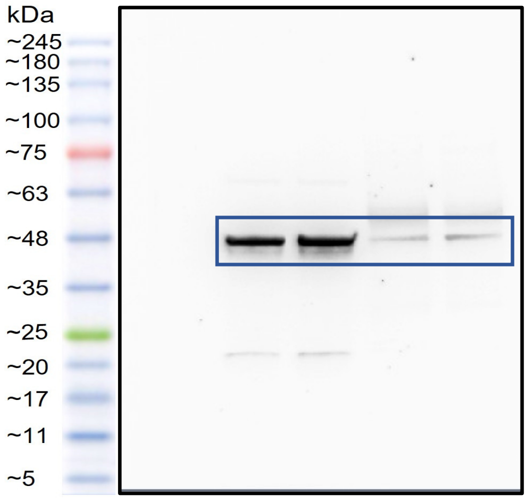

Figure 6

Fig. 6G
Myc ~48 kDa

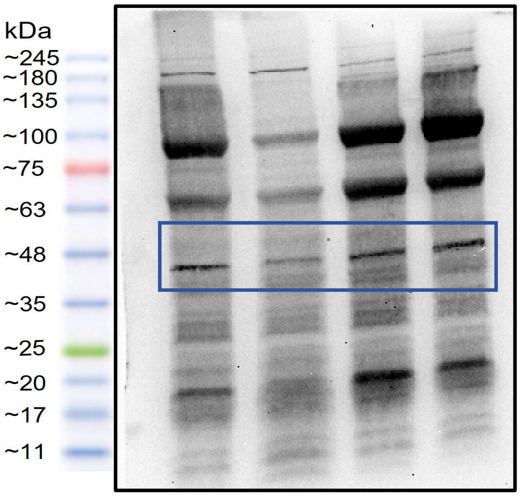

Fig. 6G
Gapdh 36 kDa

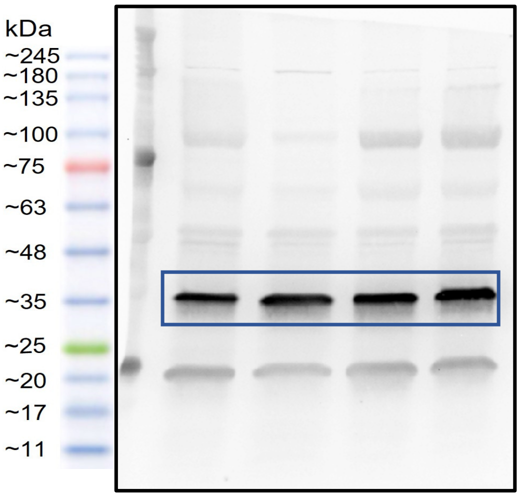

Supplementary Figure S3

Fig. S3
TERT 127 kDa

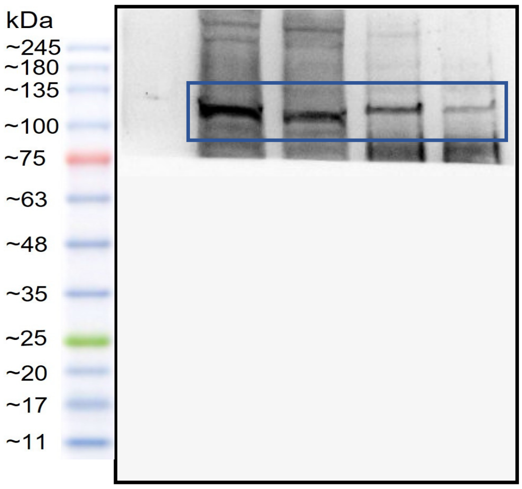

Fig. S3
p-p65 65 kDa

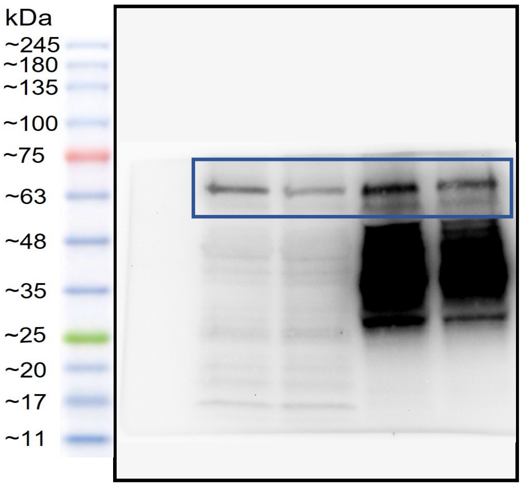

Fig. S3
α-Tubulin 50 kDa

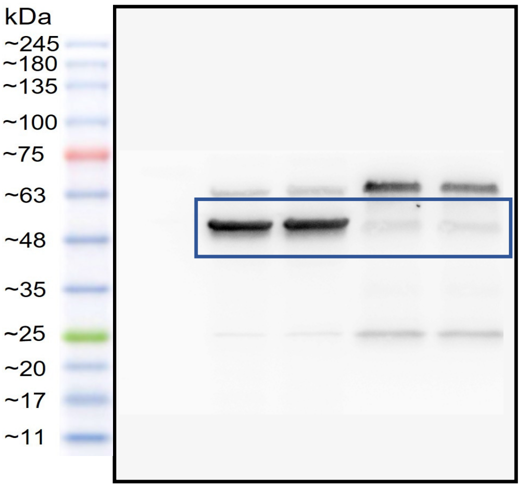

Supplementary Figure S5

Fig. S5C
TERT 127 kDa

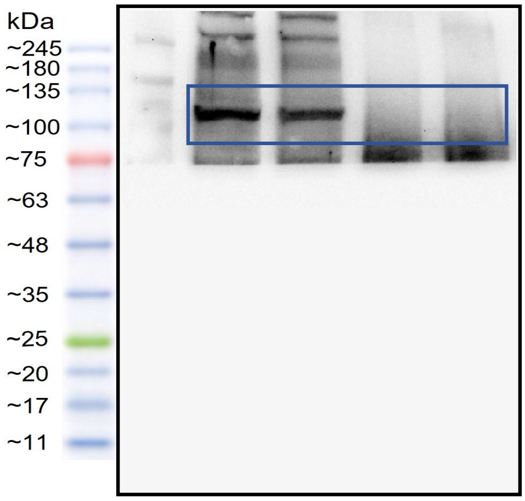

Fig. S5C
MYC 65 kDa

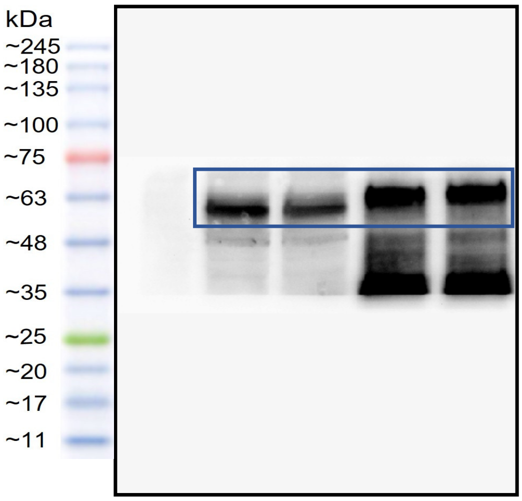

Fig. S5C
α-Tubulin 50 kDa

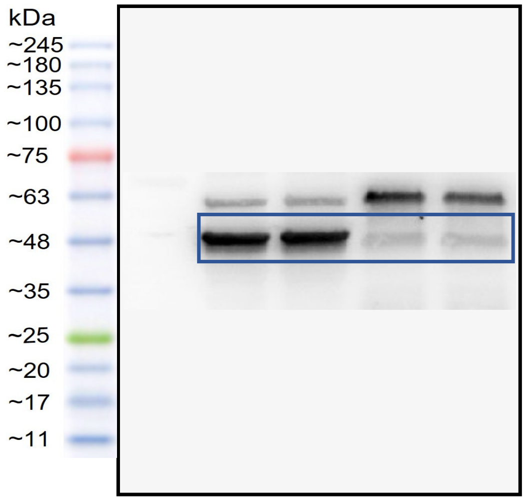

Fig. S5D
TERT 127 kDa

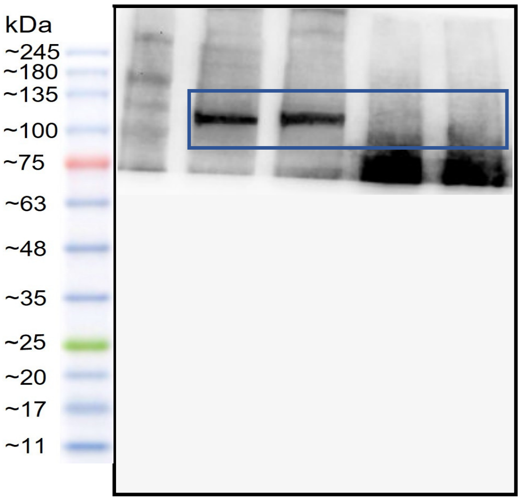

Fig. S5D
MYC 65 kDa

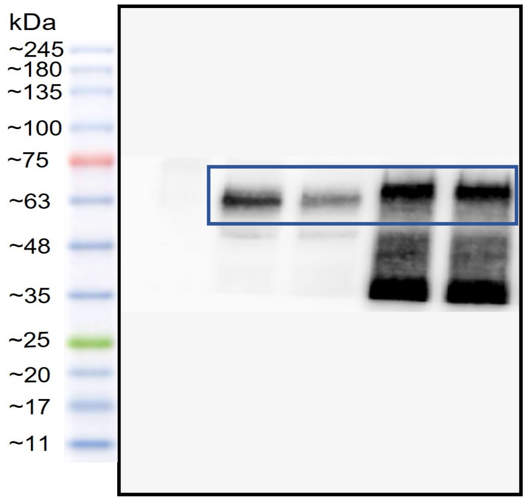

Fig. S5D
α-Tubulin 50 kDa

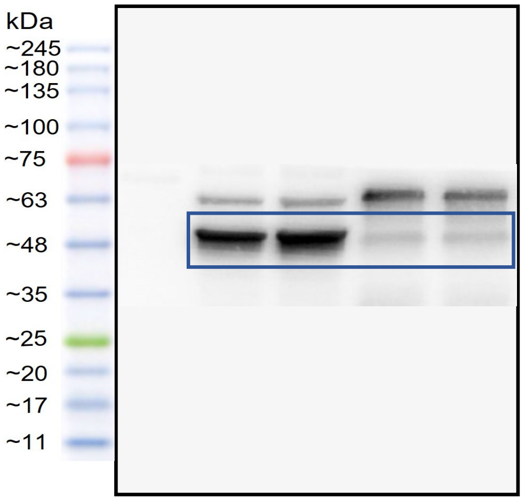

Supplement: Supplementary file 1 [file cancers-15-02673-s001.zip › File S1_Full Length Blots.pdf]
