# Supplementary material for: Short-Term TERT Inhibition Impairs Cellular Proliferation via a Telomere Length-Independent Mechanism and Can Be Exploited as a Potential Anticancer Approach
Source: Cancers (Basel). 2023 May 9;15(10):2673. doi: 10.3390/cancers15102673 (PMC10216832; doi:10.3390/cancers15102673)
Supplement: Supplementary file 1 [file cancers-15-02673-s001.zip › File S2_Supplementary Table and Figures.pdf]

Supplementary file

Short-Term TERT Inhibition Impairs Cellular Proliferation via a Telomere Length-Independent Mechanism and Can Be Exploited as a Potential Anticancer Approach

Aamir Amin [1], Marzia Morello [2], Maria Raffaella Petrara [1], Beatrice Rizzo [2], Francesco Argenton [3], Anita De Rossi [1,2,†], Silvia Giunco [1,2,*,†]

1 Department of Surgery, Oncology and Gastroenterology, Section of Oncology and Immunology, University of Padova, 35128 Padova, Italy; aamir.amin@studenti.unipd.it (A.A.); raffaella.petrara@unipd.it (M.R.P.); anita.derossi@unipd.it (A.D.R.)

2 Immunology and Diagnostic Molecular Oncology Unit, Veneto Institute of Oncology IOV—IRCCS, 35128 Padova, Italy; marzia.morello@iov.veneto.it (M.M.); beatrice.rizzo@iov.veneto.it (B.R.)

3 Department of Biology, University of Padova, 35128 Padova, Italy; francesco.argenton@unipd.it

* Correspondence: silvia.giunco@unipd.it; Tel.: +39-0498215831

† These authors contributed equally to this work.

Table S1. q-PCR primers for gene expression analysis.

**Human**		
**Gene**	**Forward Primer**	**Reverse Primer**
MYC	CCACAGCAAACCTCCTCACAG	GCAGGATAGTCCTTCCGAGTG
P21	GGAAGACCATGTGGACCTGT	GGCGTTTGGAGTGGTAGAAA
p65	AAGAGCAGCGTGGGGACTAC	CAGTGTTGGGGGCACGATTG
IκBα	CAAGCACCCGGATACAGCAG	TCAGACGCTGGCCTCCAAAC
TNFα	CTCTTCTGCCTGCTGCACTTTG	ATGGGCTACAGGCTTGTCACTC
IL6	AACCTGAACCTTCCAAAGATGG	TCTGGCTTGTTCCTCACTACT
BCL2	TGTGTGGAGAGCGTCAACCG	GGGCCGTACAGTTCCACAAA
CTNNB1	TCTGAGGACAAGCCACAAGATTACA	TGGGCACCAATATCAAGTCCAA
CCND1	CCGTCCATGCGGAAGATC	CCTCCTCCTCGCACTTCTGT
AXIN2	ATTCGGCCACTGTTCAGACG	GACAACCAACTCACTGGCCTG
Survivin	ACCGCATCTCTACATTCAAG	CAAGTCTGGCTCGTTCTC
HPRT1	TCAGGCAGTATAATCCAAAGATGGT	CTTCGTGGGGTCCTTTTCAC

**Zebrafish**		
**Gene**	**Forward Primer**	**Reverse Primer**
myca	AGCAGCAGTGGCAGCGAT-TCAGAAGATG	TGGAGAC-GTGACAGCGCTTCAAAACTAGG
mycb	AGTAG-TGACAGCGAATCCGATGACG	ATGTGGCTCTCGAATTTAATCCGC
p21	CGCAAACAGACCAACATCAC	ATGCAGCTCCAGACAGATGA
gapdh	GTGGAGTCTACTGGTGTCTTC	GTGCAGGAGGCATTGCTTACA

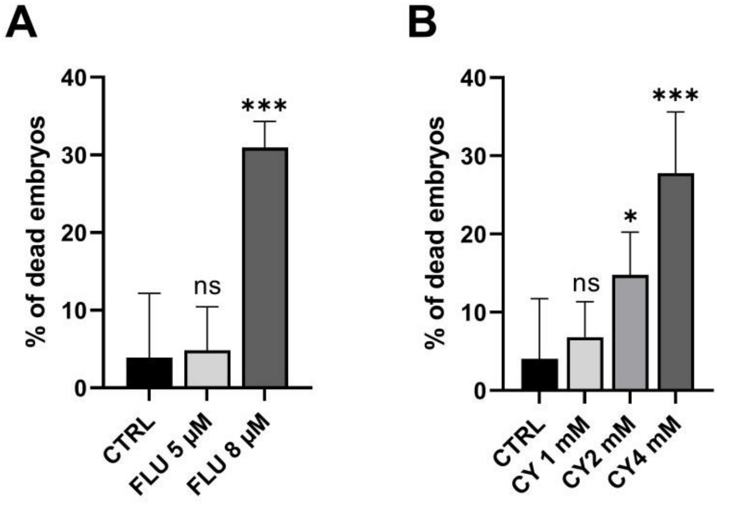

Figure S1. Effect of treatment with different doses of drugs on zebrafish embryos viability. Casper zebrafish embryos at 72 hours post-fertilization were untreated (control, CTRL) or exposed to the indicated doses of fludarabine (FLU) (**A**) or cyclophosphamide (CY) (**B**) for 72 h. Data represent the mean and SD (bar) of the percentage of dead embryos from three independent experiments with 20 embryos per group. Significant differences between values in drugs-treated embryos vs. CTRL-untreated embryos are shown: * p<0.05, *** p<0.001; ns: not significant.

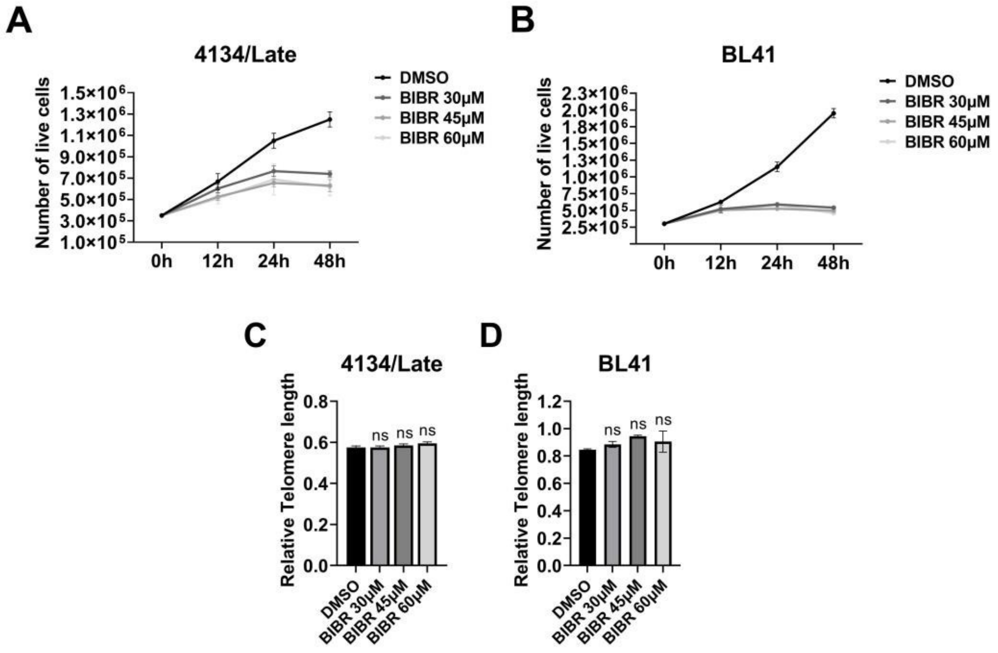

Figure S2. Effects of different doses of BIBR on proliferation rate and telomere length in LCL and BL cells. (**A**,**B**) 4134/Late (**A**) and BL41 (**B**) cells were exposed to 30, 45, or 60 µM of BIBR and the number of viable cells was analyzed by trypan blue exclusion at 12, 24 and 48 hours (h). (**C**,**D**) Relative telomere length was analyzed in 4134/L (**C**) and BL41 (**D**) cells exposed to indicated concentrations of BIBR at 24 h by multiplex real-time PCR. ns: not significant.

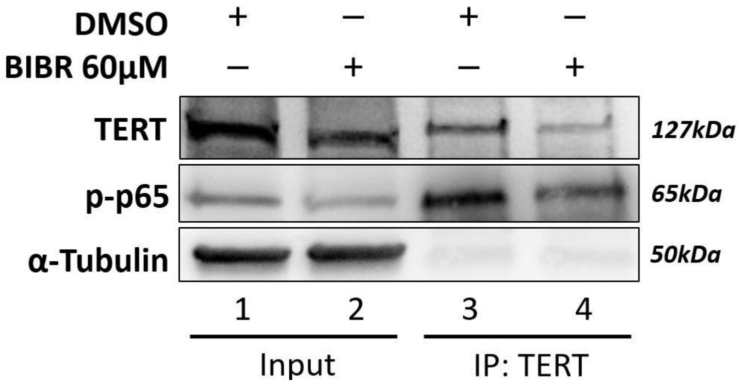

Figure S3. BIBR treatment reduced the levels of TERT/p-p65 complexes. 4134/Late cells were treated with 60 μM BIBR or DMSO for 24 h. Immunoprecipitation using 1 mg whole cell lysate was performed with TERT antibody. TERT and p-p65 were probed by Western blotting. α-tubulin was used as loading control. The original Western blots are shown in File S1.

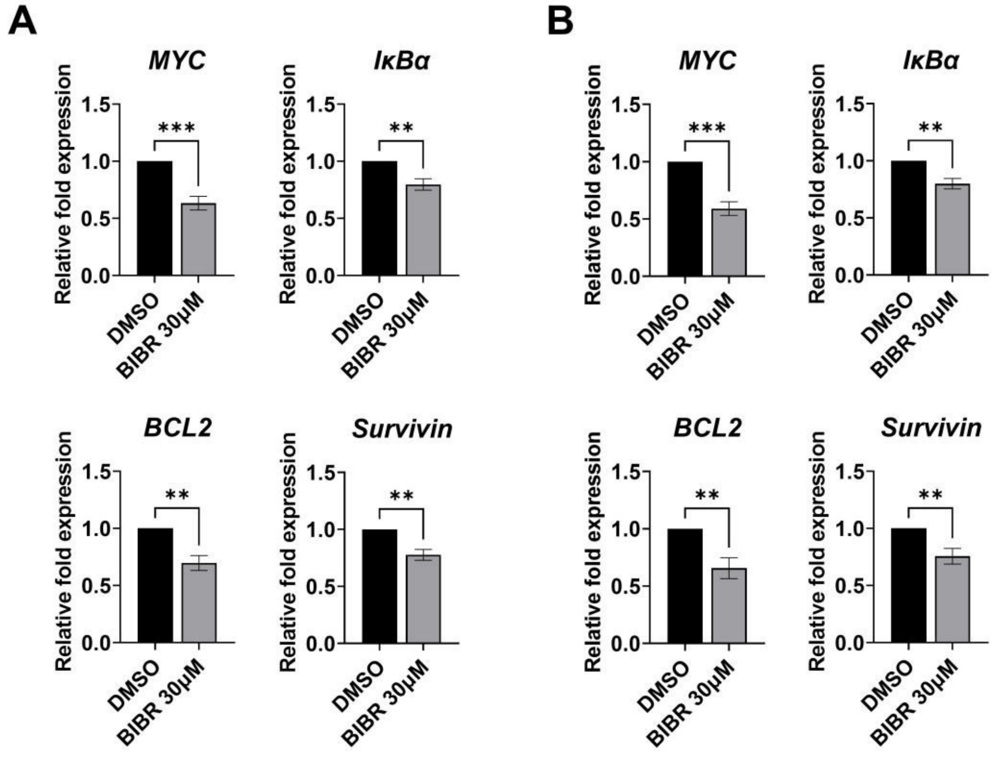

Figure S4. BIBR treatment downregulated the expression of a subset of NF-κB target genes. Cells were treated with 30 μM BIBR or DMSO as control for 24 h. Levels of relative mRNA expression for the indicated genes in 4134/Late (**A**) and BL41 (**B**) cells are shown. Data represent the mean and SD (bar) from three separate experiments. ** p<0.01; *** p<0.001.

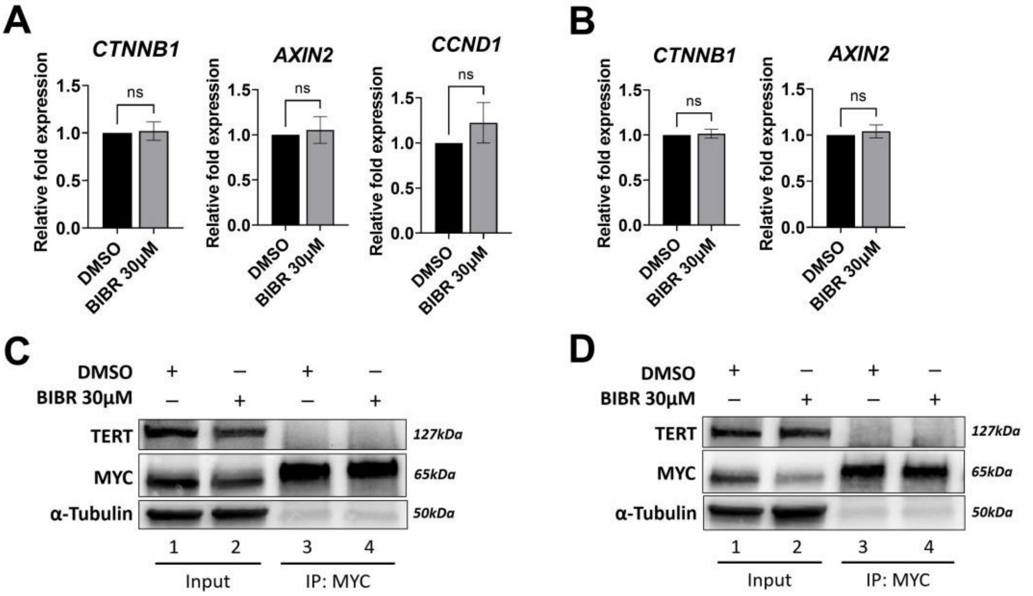

Figure S5. MYC downregulation in BIBR-treated cells is independent to WNT/β-catenin signalling. (**A**,**B**) 4134/Late (**A**) and BL41 (**B**) cells were treated with 30 µM BIBR or DMSO as control for 24 h. Levels of mRNA expression for the indicated genes are shown. Data represent the mean and SD (bar) from three separate experiments. (**C**,**D**) 4134/Late (**C**) and BL41 (**D**) cells were treated with 30 µM BIBR or DMSO for 24 h. Immunoprecipitation using 1 mg whole cell lysate was performed with MYC antibody. The indicated proteins were probed by Western blotting, and representative blots are shown. α-tubulin was used as loading control. The original Western blots are shown in File S1. ns: not significant.

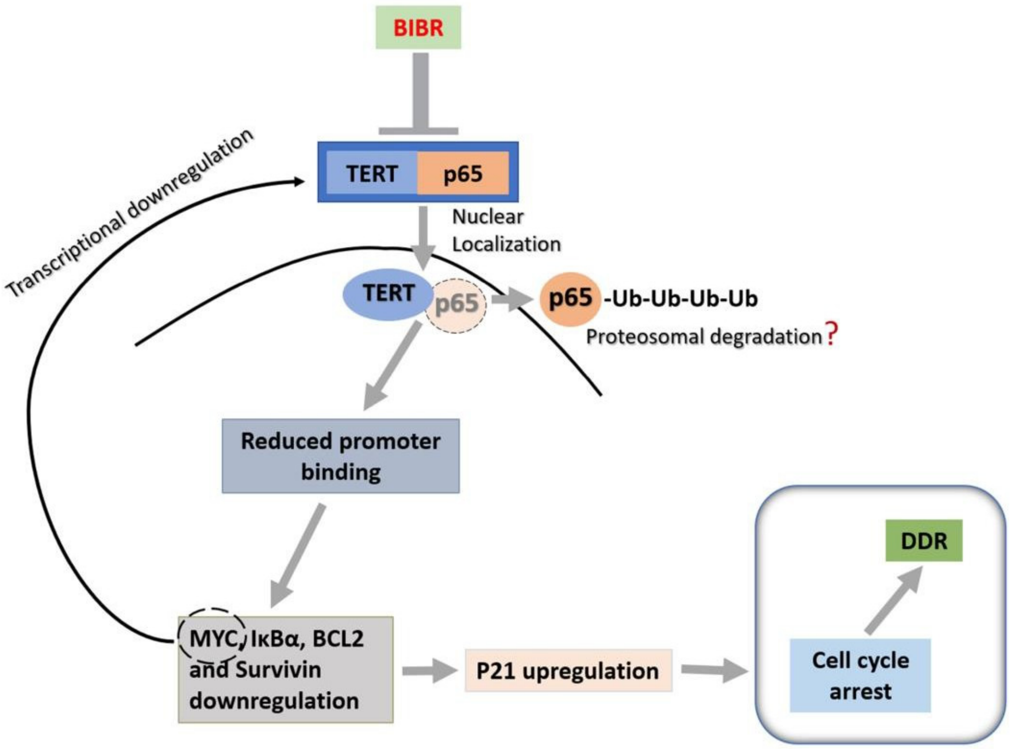

Figure S6. Schematic graph of consequences of short-term TERT inhibition in EBV-immortalized and fully transformed B cells. The TERT inhibition by BIBR downregulates NF-κB p65 nuclear localization, reducing the availability of p65 on its target promoters thereby decreasing the transcription of a subset of NF-κB p65 target genes including *MYC, IκBα, BCL2* and *Survivin*. The decreased NF-κB p65 and MYC protein levels compromise TERT promoter activation, reducing TERT expression. Furthermore, MYC downregulation compromises cellular proliferation by up-regulating P21 expression and its nuclear localization thereby leading to the S-phase cell cycle arrest which may ultimately contribute to the activation of telomere length-independent DDR mediated by replication fork stalling.
